# A review of the taxonomic diversity, host–parasite interactions, and experimental research on chytrids that parasitize diatoms

**DOI:** 10.3389/fmicb.2023.1281648

**Published:** 2023-10-30

**Authors:** August Danz, C. Alisha Quandt

**Affiliations:** ^1^Department of Ecology and Evolutionary Biology, University of Colorado, Boulder, CO, United States; ^2^University of Colorado Museum of Natural History, Boulder, CO, United States

**Keywords:** diatoms (Bacillariophyceae), fungi, diversity, parasitism, mycoloop, mechanism, infection, freshwater

## Abstract

Diatoms (Bacillariophyta) are a major source of primary production on Earth, generating between 1/4 to 1/2 of all oxygen. They are found in almost all bodies of water, the ice of mountains, the arctic and the antarctic, and soils. Diatoms are also a major source of food in aquatic systems, a key component of the silica cycle, and are carbon capturers in oceans. Recently, diatoms have been examined as sources of biofuels, food, and other economic boons. Chytrids are members of the Kingdom fungi comprising, at a minimum, Chytridiomycota, Blastocladiomycota, and Neocallimastigales. Most chytrids are saprobes, plant pathogens, or parasites, and play an important role in aquatic ecosystems. Chytrid parasitism of diatoms has been reported to cause epidemics of over 90% fatality, though most of the information regarding these epidemics is limited to interactions between just a few hosts and parasites. Given the ubiquity of diatoms, their importance in natural and economic systems, and the massive impact epidemics can have on populations, the relative lack of knowledge regarding parasitism by chytrids is alarming. Here we present a list of the firsthand accounts of diatoms reported parasitized by chytrids. The list includes 162 named parasitic chytrid-diatom interactions, with 63 unique chytrid taxa from 11 genera, and 74 unique diatom taxa from 28 genera. Prior to this review, no list of all documented diatom-chytrid interactions existed. We also synthesize the currently known methods of infection, defense, and experiments examining diatoms and chytrids, and we document the great need for work examining both a greater breadth of taxonomic diversity of parasites and hosts, and a greater depth of experiments probing their interactions. This resource is intended to serve as a building block for future researchers studying diatom-parasite interactions and global planktonic communities in both fresh and marine systems.

## Introduction

### Diatoms

Diatoms (Bacillariophyta) are a major source of primary production and oxygen at both local and global scales (Mann, 1999; Hassett and Gradinger, 2016). They are estimated to generate between ¼ to ½ of all oxygen on earth (Scholz et al., 2016a; Pančić et al., 2019; Grønning and Kiørboe, 2020). In addition, diatoms are also a major source of food for zooplankton, and a major component of silica sequestration in freshwater systems and oceans (Sommer, 1987; Smetacek, 1999; Julius and Theriot, 2010; Smetacek, 2018).

**Figure 1:**
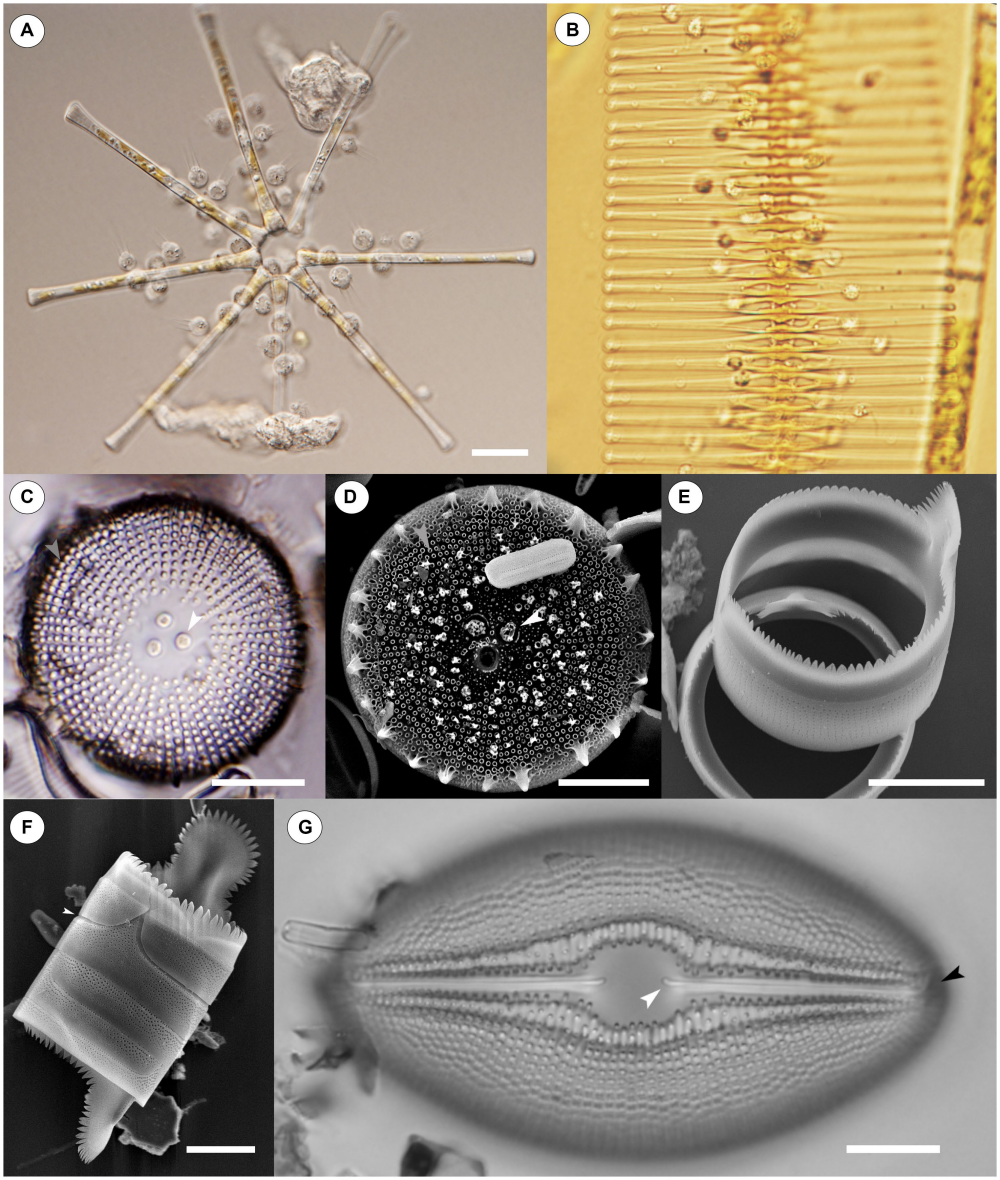
Light Microscope (LM) and Scanning Electron Microscope (SEM) images of chytrids infecting diatoms and diatoms on their own. **(A)** Numerous chytrids encysted on a stellate colony of *Asterionella formosa.* Scale = 20 μM. **(B)** Chytrids infecting a chain of *Fragilaria crotonensis.* Scale = not shown. **(C)** An LM image of *Orthoseira* sp., the white central arrow points to carinoportulae, a special pore like structure found only within the family *Orthoseiraceae*, while the light gray arrow identifies areolae in striae. Scale = 10 μM. **(D)** An SEM image of *Orthoseira* sp., the white arrow again identifies carinoportulae while the light gray arrow points to areolae. Areolae are a common ornamentation found on diatoms and facilitate material transfer through the glass frustule; they are also a proposed point of entry into the glass frustule for chytrid parasites. Scale = 10 μM. **(E)** A girdle band resting atop the edge of another girdle band. Some poration can be seen along the sides of each band. Scale = 10 μM. **(F)** Four girdle bands attached together without valves. The top girdle band has been slightly dislodged from the rest (white arrow); the joints between girdle bands are a proposed area chytrids bypass the protection of the frustule. Scale = 10 μM. **(G)** An LM image of *Diploneis* sp. The proximal end of one raphe is indicated by a white arrow while the distal end of the raphe is indicated by a black arrow. Scale = 10 μM. Raphe are another proposed point of entry for chytrid parasites. Images for **(A,B,G)** courtesy of Dr. J.P. Kociolek.

Diatoms evolved somewhere in the early Mesozoic era, around 250 million years ago (Mekhalfi et al., 2014). They are single celled, eukaryotic organisms, often found free floating or growing in filamentous colonies (Round et al., 1990). These organisms are most well known for their siliceous cell wall, termed the frustule (Round et al., 1990; Spaulding et al., 2021). The frustule can be divided into two halves, or theca, of which one is larger (epitheca) and the other is smaller (hypotheca) (Round et al., 1990; Julius and Theriot, 2010; Spaulding et al., 2021). Each theca of a diatom frustule can also be divided into two main parts, the valve, and the cingulum (Figures 1C–F). The valve refers to the siliceous unit at the end of the theca where a subsequent cell may attach to form a chain (Spaulding et al., 2021). The cingula is the series of siliceous copulae, colloquially referred to as bands, which extend away from the valves. These bands overlap one another, and help increase the interior volume of cells (Round et al., 1990).

Since diatoms are encased by the frustule, effectively a glass suit of armor, cells are limited in their ability to interact with the environment (Round et al., 1990). As a result, the frustule has a myriad of openings, sometimes referred to as ornamentation, that allow cells to pass materials in and out of the glass shell, as well as interact with their local environment (Julius and Theriot, 2010). These features include a variety of specialized pores on both the valve and the girdle (areolae, porelli, carinoportulae, rimoportulae, etc.), spines, striation patterns, symmetry, and the raphe which can be found in some pennate diatoms and allow for movement (Round et al., 1990) (Figures 1C–G). Diatom taxonomy has largely centered around these morphological features of the frustule, predominantly those of the valves (Julius and Theriot, 2010). Diatoms can be found in nearly all bodies of water in the world, in the ice on mountaintops in the arctic and antarctic, and growing on soils and other aerophilous habitats (Douglas and Smol, 2001; Julius and Theriot, 2010; Kociolek et al., 2017; Danz et al., 2022).

### Chytrids

Chytrids are fungi that have a motile zoospore with an opithokont flagellum. They produce one to many sporangia inside (endobiotic) or outside (epibiotic) of their substrate or host, and may produce rhizoids, which are anucleate, filamentous, root-like structures acting as a feeding organ (Kirk et al., 2008). Several lineages within Kingdom Fungi are considered chytrids (depending on which classification system is used) but at a minimum comprise Chytridiomycota Arx, Blastocladiomycota T. Y. James, and Neocallimastigales J.L. Li, I.B. Heath and L. Packer (Spatafora et al., 2017). Ecologically, most chytrids are saprobes, plant pathogens, parasites of other fungi and protists, or algal parasites. The importance of chytrids in aquatic food webs is broadly recognized in the so-called mycoloop (Kagami et al., 2007a).

Much of the early work on chytrids centered on their basic description and identification (Sparrow, 1960). But much of the diversity that has been described is from Europe and North America due to the historical prevalence of researchers in those places. It is likely that most of their diversity remains undescribed, and many biogeographical questions are currently unanswerable due to a lack of true distributional data and the force fitting of European names on a global scale. A number of detailed reviews surrounding chytrids and their role in plankton ecology already exist (Ibelings et al., 2004; Frenken et al., 2017); however, no review has focused on the parasitic relationship between chytrids and diatoms, until now.

### Known interactions between chytrid parasites and diatom hosts

Considerable investigations into grazers of diatoms (Anderson et al., 1955; Geller, 1975; Lampert and Schober, 1978; Geller, 1980; Reynolds et al., 1982; Sommer, 1984; Lehman and Sandgren, 1985; Bruning, 1991b; Holfeld, 1998; Ibelings et al., 2004; Pančić et al., 2019; Grønning and Kiørboe, 2020), as well as the evolutionary adaptations diatoms have developed in response to these threats, exist (Patrick, 1977; Reynolds et al., 1982; Smetacek, 1999; Kagami et al., 2007a, 2014). Less, but still a substantial amount, is known regarding the role aquatic parasites (namely the Chytridiomycota but also the Oomycetes G. Winter and other protists) play in phytoplankton networks (Bruning, 1991a,b; Holfeld, 1998; Ibelings et al., 2004; Rad-Menéndez et al., 2018) especially in freshwater lentic environments (Canter, 1947, 1950a,b, 1951, 1953, 1954, 1967, 1969, 1970, 1979; Canter and Lund, 1948; Canter and Lund, 1951a; Canter and Jaworski, 1978, 1979, 1980, 1981, 1982, 1986; Sommer, 1987; Bruning, 1991a,b,c,d; Van Donk and Bruning, 1992; Bertrand et al., 2004; Kagami et al., 2007a, 2012; Maier and Peterson, 2014; Scholz et al., 2017a). Indeed, a simple search on the web of science for “diatom grazers” or “grazers of diatoms” yields 581 results, while a search for “parasites of diatoms” or “diatom parasites” yields 266 and 270 results, respectively, [5 March 2023].

Among the published studies of chytrid parasites of diatoms, much of the work covers the population effects fungal parasites have on diatoms, with epidemics causing whole population collapses — in some cases reducing host populations by 90%, dictating seasonal variations and abundance, and opening niches for smaller, less competitive species (Canter and Lund, 1948; Reynolds, 1972; Sommer, 1987; Bruning, 1991c; Van Donk and Bruning, 1992; Canter-Lund and Lund, 1995; Ibelings et al., 2004; Kagami et al., 2007a; Rad-Menéndez et al., 2018; Reñé et al., 2022). Other work has examined the impacts abiotic factors such as light/darkness, temperature, ice cover and hydrodynamic disturbance, as well as biotic factors relating to host specificity and cell/colony shape, have on rates and prevalence of infection (Reynolds, 1972; Canter and Jaworski, 1978, 1981, 1982; Van Donk and Ringelberg, 1983; Bruning, 1991a,b; Canter et al., 1992; Holfeld, 1998; Bertrand et al., 2004). More recently, efforts to tie molecular data to species binomials has been undertaken, but this is an area needing greater study (Rad-Menéndez et al., 2018).

A substantial amount of work has been reported recently discussing the role fungal parasites of diatoms, and phytoplankton in general, has in aquatic food webs. Assessments based on DNA barcoding estimate that fungal parasites are dominant in some bodies of water (Jobard et al., 2010; Reñé et al., 2022). They can comprise a large portion of all eukaryotic genetic material in some aquatic systems, and they may be a major component of food web cycling due to particlization and break down of inedible contents (large diatoms) into grazable material; this has been termed the mycoloop or the F-Z link (Kagami et al., 2007a,b; Gleason et al., 2008; Grami et al., 2011; Miki et al., 2011; Kagami et al., 2012; Sime-Ngando, 2012; Kagami et al., 2014; Cleary and Durbin, 2016; Rad-Menéndez et al., 2018; Grossart et al., 2019). This pathway has been reported in both fresh and marine environments (Hassett and Gradinger, 2016). The discovery of the mycoloop, and its potential impact on nutrient cycling indicates the importance of fungal-algal interactions in natural systems. Chytrids have also been reported to stabilize food webs, while also reducing the amount of organic material that reaches benthic environments (Grami et al., 2011).

With the increasing prevalence of ‘omics-based techniques, work has begun to identify chytrids, and chytrid-diatom interactions, using molecular methods. These efforts are still in early stages (Maier and Peterson, 2014), but already many undescribed taxa of chytrids are now known to exist (Seto et al., 2020). Formal classification, mainly relying on detailed microscopic observations, is still required to describe these organisms. There is also no current way to determine if a chytrid is parasitizing a diatom by exclusively using these methods, but some inferences can be made based on timing, taxa, and the amount of host and parasite present in conjunction with live microscopy and single cell ‘omics. Also stemming from this work is a better understanding of the phylogeny of the Chytridiomycota (Seto et al., 2023).

### Areas requiring further research

Despite this substantial amount of research, critical understanding of these aquatic fungal parasites is missing, as there are few detailed accounts describing how fungal parasites attack diatoms—particularly in regards to the initial infection, and much of the experimental work examining chytrid outbreaks on diatoms has been limited to a few host species, namely *Asterionella* Hass (Figure 1A). Kagami et al. (2014) reports there are “more than 700 species of chytrids known to infect phytoplankton, zooplankton, fungi, plants, and invertebrate animals” and Ibelings et al. (2004) comments: “It is probably fair to say that every species is affected by parasites in one way or another. Hence, parasites must be abundant in the phytoplankton too, although not much is known.” Letcher and Powell (2012) identify over 220 species in the genus *Rhizophydium* Schenk ex Rabenh. alone. With over 75,000 taxa of diatom reported (Kociolek et al., 2018: Kociolek, 2019) and 1,250 species of chytrid (Shearer et al., 2007), combined with the wide impacts both organism types have in aquatic systems, we anticipate that there are many more parasitic interactions occurring than currently reported (Scholz et al., 2016a). A larger number of interactions is especially likely when considering the gaps in the current understanding of chytrid systematics (Seto et al., 2016, 2020). Furthermore, when compared to the upwards of 200,000 estimated species of diatom (Guiry, 2012; Mann and Vanormelingen, 2013), and the estimates of somewhere between 1.5 million to 3.5 million total fungal species—with approximately 150,000 described so far (Hyde, 2022)—it is easy to assume that there are more diatom-chytrid interactions occurring. Scientific interest regarding chytrids, and fungal parasites as a whole, has increased recently, likely as a result of the unexpected diversity now known to be present within the group and the emergence of novel infectious diseases (Frenken et al., 2017; Fisher et al., 2020).

There are a few reasons why current knowledge may be limited. Historically, these parasites have been understudied due to difficulties with identification, quantification, and isolation, though the use of dyes can make observation of chytrids infecting hosts easier (Sparrow, 1960; Johnson, 1966b; Canter, 1979; Sommer, 1987; Holfeld, 1998; Holfeld, 2000a,b; Kagami et al., 2012; Voigt et al., 2013; Scholz et al., 2016a). Furthermore, observing all of the stages of a chytrid’s life cycle may be needed to identify an individual to the species level, which is not always possible in natural environments (Sen, 1989). Exacerbating this, few researchers have focused on the description of novel chytrid parasites of diatoms (Kagami et al., 2007a; Voigt et al., 2013), with a majority of the studied combinations having been made in the early to mid 1900s (See Table 1). A lack of biomass, as Kagami et al. (2007b) puts it, or the low background level of infections as Maier and Peterson (2014) identified, has also led to researchers ignoring these groups.

**Table 1:** Diatom hosts with firsthand accounts of a chytrid parasite, the habitat they are found in, the locality they are from, and all references documenting the interaction.

Host [reported name if different]	Parasite [reported name if different]	Habitat	Reported locality	Reference(s)
*Achnanthidium affine* (Grunow) Czarnecki [*Achnanthes affinis* Grunow]	*Rhizophydium achnanthis* Friedmann	Freshwater	Floridsdorfer Wasserpark	Friedmann (1952)
*Amphora ovalis* (Kützing) Kützing	*Physorhizophidium pachydermum* Scherffel	Freshwater	Hungary	Scherffel (1926), Karling (1977)
*Amphora proteus* W.Gregory	cf. *Podochytrium clavatum* Pfitzer	Marine	Core Creek, Carteret County, North Carolina	Johnson (1966b)
*Amphora* sp.	*Podochytrium clavatum* Pfitzer	Freshwater	Hungary	Scherffel (1926)
*Asterionella formosa* Hass	*Chytriomyces* sp.	Freshwater		Canter and Lund (1953)
	*Rhizophidium tetragenum* Pongratz	Freshwater	Lake Geneva, Switzerland; Schöhsee Lake near Plon, North Germany	Pongratz (1966), Karling (1977), Holfeld (1998, 2000b)
	*Rhizophydium planktonicum* Canter	Freshwater	English Lake District; Lake Maarseveen?; Douglas Lake, Base Lake, Portage Lake Michigan	Canter and Lund (1948, 1951b, 1953), Canter (1953), Paterson (1958b), Koob (1966), Canter (1969), Canter and Jaworski (1978, 1979, 1980, 1981), Bruning and Ringelberg (1987), Kudoh and Takahashi (1990), Bruning (1991a,b,c,d), Beakes et al. (1993), Doggett and Porter (1996), Holfeld (1998, 2000b), Rasconi et al., 2012, Seto et al. (2016), Kagami et al. (2020)
	*Rhizophydium schroeteri* De Wildeman	Freshwater	Lake Geneva, Switzerland	Pongratz (1966)
	*Rhizophydium* sp.	Freshwater	Grand traverse Bay, Lake Michigan	Paterson (1967)
	*Septosperma anomala* (Couch) Whiffen ex Seym	Freshwater		Canter and Lund (1953)
	*Zygophlyctis asterionellae* Seto [*Zygorhizidium melosirae* Canter]	Freshwater	Grand Traverse Bay, Lake Michigan	Paterson (1967), Karling (1977)
	*Zygophlyctis asterionellae* Seto [*Zygorhizidium planktonicum* Canter]	Freshwater	Many: Lake Maarsveen; English Lake District; Pond Biwa, Nagano, Japan	Canter and Lund (1953), Canter (1967, 1979), Van Donk and Ringelberg (1983), Canter and Jaworski (1982, 1986), Beakes et al. (1988), Van Donk (1989), Beakes et al. (1992, 1993), Holfeld, (1998, 2000b), De Bruin et al. (2004), Kagami et al. (2004, 2005, 2007b), Gsell et al. (2013b), Maier and Peterson (2014), Seto et al. (2016, 2020)
	*Zygorhizidium affluens* Canter	Freshwater		Canter and Lund (1953), Canter (1969, 1979), Reynolds (1972), Karling (1977), Youngman et al. (1976), Sen (1987a), Beakes et al. (1988, 1992, 1993), Kudoh and Takahashi (1990), Holfeld (1998, 2000b), Rad-Menéndez et al. (2018)
	*Zygorhizidium asterionellae* Pongratz	Freshwater	Lake Geneva, Switzerland	Pongratz (1966)
*Asterionella formosa* var. *gracillima* (Hanztsch) Grunow [*Asterionella gracillima* (Hanztsch) Heiberg]	*Rhizophydium schroeteri* De Wildeman	Freshwater	Lac de Zurich, Switzerland	De Wildeman (1900, 1931)
*Asterionella* sp.	*Zygophlyctis melosirae* (Canter) Seto [*Zygorhizidium melosirae* Canter]	Freshwater	Frains and Silver Lakes, Washtenaw County, Michigan	Paterson (1958a)
*Aulacoseira ambigua* (Grunow) Simonsen	cf. *Zygorhizidium planktonicum* Canter	Freshwater	Lake Inba, Japan	Kagami et al. (2012)
	*Zygophlyctis* aff. *Melosirae* (Canter) Seto [*Zygorhizidium* aff. *Melosirae* Canter]	Freshwater	Lakes in Japan	Seto et al. (2016)
	*Zygophlyctis melosirae* (Canter) Seto [*Zygorhizidium melosirae* Canter]	Freshwater	Lake Inba, Japan	Kagami et al. (2020), Seto et al. (2020)
*Aulacoseira ambigua* (Grunow) Simonsen [*Melosira ambigua* (Grunow) Müller]	cf. Zygorhizidium affluens Canter	Freshwater	Shearwater	Sen (1989)
	*Chytridium melosirae* Sparrow	Freshwater	Sweden	Sparrow (1960)
	*Chytridium versatile* Scherffel	Freshwater	Sweden	Skuja, (1948)
	*Rhizophydium fusus* (Zopf) Fischer	Freshwater	Sweden	Skuja (1948)
*Aulacoseira granulata* (Ehrenberg) Simonsen	cf. *Zygophlyctis melosirae* (Canter) Seto [cf. *Zygorhizidium melosirae* Canter]	Freshwater	Lake Inba, Japan	Kagami et al. (2012)
	Rhizophydiales sp.	Freshwater	Lake Inba, Japan	Kagami et al. (2020)
	*Zygophlyctis* aff. *Melosirae* (Canter) Seto [*Zygorhizidium* aff. *Melosirae* Canter]	Freshwater	Lakes in Japan	Seto et al. (2016)
*Aulacoseira granulata* (Ehrenberg) Simonsen [*Melosira granulata* (Ehrenberg) Ralfs]	*Rhizophydium fusus* (Zopf) Fischer	Freshwater		Canter and Lund (1953)
	*Zygophlyctis melosirae* (Canter) Seto [*Zygorhizidium melosirae* Canter]	Freshwater	Provo Bay, Utah Lake, Utah	Felix (1977)
*Aulacoseira islandica* (Otto Müller) Simonsen [*Melosira islandica* Otto Müller]	*Zygophlyctis melosirae* (Canter) Seto [*Zygorhizidium melosirae* Canter]	Freshwater	Lake Geneva, Switzerland	Pongratz (1966)
*Aulacoseira italica* (Ehrenberg) Simonsen [*Melosira italica* (Ehrenberg) Kutzing]	*Rhizophydium pedicellatum* Paterson	Freshwater	South Fish Tail Bay, Douglas Lake, Cheboygan County, Michigan	Paterson (1956)
	*Septosperma* sp.	Freshwater		Canter and Lund (1953)
	*Zygophlyctis melosirae* (Canter) Seto [*Zygorhizidium melosirae* Canter]	Freshwater	Esthwaite Water (type locality), Windermere, Blelham Tarn and Ullswater in the English Lake District	Canter, (1950a), Canter and Lund (1951a, 1953), Canter (1953, 1967), Karling (1977), Doggett and Porter (1996), Seto et al. (2020)
*Cocconeis pediculus* Ehrenberg	*Chytridium cocconeidis* Canter	Freshwater	Bradbourne Park Lake, Sevenoaks, Kent, England	Canter (1947), Karling (1977)
*Coscinodiscus* sp.	*Rhizophydium* sp.	Freshwater	Grand traverse Bay, Lake Michigan	Paterson (1967)
*Cyclotella chaetoceras* Lemmermann	*Rhizophydium cyclotellae* Zopf			Domjan (1936)
*Cyclotella* sp.	*Rhizophydium cyclotellae* Zopf	Freshwater		Sparrow (1960), Rasconi et al. (2012)
*Cymatopleura elliptica* (Brébisson) W.Smith	*Rhizophydium clinopus* Scherffel			Scherffel (1930), Sparrow (1960)
*Cymbella aspera* (Ehrenberg) Cleve	*Rhizophydium clinopus* Scherffel	Freshwater	Gaaden, Austria	Friedmann (1952)
*Cymbella* sp.	*Chytridium perniciosum* Sparrow	Freshwater	Floridsdorfer Wasserpark	Friedmann (1952)
	*Rhizophydium clinopus* Scherffel			Scherffel (1930), Sparrow (1960)
	*Rhizophydium fusus* (Zopf) Fischer		Hungary	Scherffel (1902)
*Diatoma* sp.	*Rhizophydium schroeteri* De Wildeman	Freshwater	Lake Geneva, Switzerland	Pongratz (1966)
*Discoplea rotula* Ehrenberg [*Stephanodiscus rotula* (Kützing) Hendey]	*Podochytrium cornutum* Sparrow	Freshwater	Crosemere, Cheshire	Canter (1970)
	*Rhizophydium cyclotellae* Zopf	Freshwater	Loch Leven, Scotland	Bailey-Watts and Lund (1973)
*Epithemia adnata* (Kützing) Brébisson [*Epithemia zebra* (Ehrenberg) Kützing]	*Chytridium epithemiae* Nowakowski	Freshwater		Karling (1977)
	*Rhizophydium epithemiae* Valkanov		Bulgaria	Sparrow (1960)
*Eunotia* sp.	*Rhizophydium clinopus* Scherffel	Freshwater	Blue Pond, Ellington Drive, Muirkirk, Prince Georges County, Maryland	Paterson (1962)
*Fragilaria capucina* Desmazières	*Rhizophydium fragilariae* Canter	Freshwater	Portage Lake, Washtenaw County, Michigan	Paterson (1958b)
*Fragilaria crotonensis Kitton*	*Chytridium versatile* Scherffel	Freshwater		Canter (1950a, 1953), Canter and Lund (1953), Rasconi et al. (2012)
	*Rhizophidium tetragenum* Pongratz	Freshwater	Schöhsee Lake near Plon, North Germany	Holfeld (1998, 2000b)
	*Rhizophydium fragilariae* Canter	Freshwater	English Lake District; Shearwater, Ireland; Lake Constance; Portage Lake, Washtenaw County, Michigan; Grand Traverse Bay, Lake Michigan; Lake Geneva, Switzerland	Canter (1950a, 1953), Canter and Lund (1953), Paterson (1958b, 1967), Sparrow (1960), Pongratz (1966), Canter and Jaworski (1982), Sommer (1984), Sen (1987b), Rasconi et al. (2012)
	*Rhizophydium planktonicum* Canter	Freshwater		Canter and Lund (1951b)
	*Zygorhizidium* sp.	Freshwater	Schöhsee Lake near Plon, North Germany	Holfeld (1998¸ 2000b)
*Fragilaria* sp.	*Podochytrium clavatum* Pfitzer	Freshwater	Surrey; Great Britain	Sparrow (1936a), Canter (1953)
	*Rhizophydium schroeteri* De Wildeman	Freshwater	Lake Geneva, Switzerland	Pongratz (1966)
	*Rhizophydium* sp.	Freshwater	Grand traverse Bay, Lake Michigan	Paterson (1967)
*Gomphonema micropus* Kützing	*Podochytrium clavatum* Pfitzer	Freshwater	Hungary	Scherffel (1926)
*Gomphonema truncatum* Ehrenberg [*Gomphonema constrictum* Ehrenberg]	*Rhizophydium fusus* (Zopf) Fischer		Hungary	Scherffel (1902)
*Hantzschia amphioxys* (Ehrenberg) Grunow [*Eunotia amphioxys* Ehrenberg]	*Rhizophydium globosum* (Braun) Rabenhorst	Freshwater		Sparrow (1960)
	*Rhizophydium globosum* (Braun) Rabenhorst [*Chytridium globosum* (Braun) Rabenhorst]	Freshwater		Braun (1855, 1856), Sparrow (1960)
*Melosira nummuloides* Agardh	*Chytridium anatropum* Braun [cf. *Phlyctidium anatropum* (Braun) Sparrow]	Marine	Core Creek, Carteret County, North Carolina	Johnson (1966b)
*Melosira hummii* Hustedt	*Rhizophydium fusus* (Zopf) Fischer	Marine	Flanner’s Beach, Neuse River, North Carolina	Johnson (1966b)
	*Rhizophydium globosum* (Braun) Rabenhorst	Marine	Adams Creek at the Neuse River, North Carolina	Johnson (1966b)
*Melosira* sp.	cf. *Rhizophydium melosirae* Friedmann	Freshwater	Portage Lake, Washtenaw County, Michigan	Paterson (1958c)
	*Chytridium versatile* Scherffel	Freshwater	Sweden	Skuja (1948)
	*Podochytrium emmanuelense* (Sparrow) Sparrow and Paterson	Freshwater	United States	Brodie et al. (1955)
	*Rhizophydium distinctum* Petersen			Peterson (1905)
	*Rhizophydium fusus* (Zopf) Fischer	Freshwater	Jardin Botanique de Nancy	De Wildeman (1894), Litvinow (1953)
	*Rhizophydium globosum* (Braun) Rabenhorst	Freshwater	Jardin Botanique de Nancy	De Wildeman (1894)
	*Rhizophydium marinum* de Wildeman	Marine		De Wildeman (1890)
	*Septocarpus corynephorus* Zopf	Freshwater	Jardin Botanique de Nancy	De Wildeman (1894)
	*Zygophlyctis melosirae* (Canter) Seto [*Zygorhizidium melosirae* Canter]	Freshwater	Frains and Base Lakes, Washtenaw County, Michigan	Paterson (1958a)
*Melosira varians* Agardh	*Chytridium adpressum* Sparrow [*Chytridium appressum* Sparrow]	Freshwater	Fall Creek in Forest Home, New York; Cambridge	Sparrow (1933b, 1936a), Canter (1953), Karling (1977)
	*Chytridium epithemiae* Nowakowski	Freshwater	Fall Creek in Forest Home, New York	Sparrow (1933b)
	*Chytridium lagenula* Braun	Freshwater	Dreisam River, Near Freiberg, Germany	Braun (1855, 1856)
	*Chytridium nodulosum* Sparrow	Freshwater	United States	Sparrow (1943, 1960)
	*Chytridium versatile* Scherffel	Freshwater	Hungary	Domjan (1936)
	*Podochytrium clavatum* Pfitzer	Freshwater	Floridsdorfer Wasserpark; Hungary	Scherffel (1926), Friedmann (1952)
	*Podochytrium emmanuelense* (Sparrow) Sparrow and Paterson	Freshwater		Karling (1977)
	*Podochytrium emmanuelense* (Sparrow) Sparrow and Paterson [*Rhizidiopsis emmanuelensis* Sparrow]	Freshwater	Cambridge	Sparrow (1933a, 1936a), Canter (1953)
	*Podochytrium lanceolatum* Sparrow	Freshwater	Cambridge	Sparrow (1933a, 1936a, 1951), Canter (1953), Karling (1977)
	*Podochytrium* sp.	Freshwater	Lake Lanier, Georgia	Doggett and Porter (1996)
	*Rhizophydium fusus* (Zopf) Fischer	Freshwater	Coe Fen, Cambridge and Surrey; Hungary; Fall Creek, and Near Cold Spring Harbor, New York	Sparrow (1932, 1933c), Canter (1953)
	*Rhizophydium globosum* (Braun) Rabenhorst	Freshwater		Sparrow (1960)
	*Rhizophydium globosum* (Braun) Rabenhorst [*Chytridium globosum* (Braun) Rabenhorst]	Freshwater	Jardin Botanique de Nancy	Braun (1855, 1856), De Wildeman (1890)
	*Rhizophydium lagenula* (Braun) Fischer	Freshwater	Coe Fen, Cambridge	Sparrow (1936a)
	*Rhizophydium melosirae* Friedmann	Freshwater	Floridsdorfer Wasserpark	Friedmann (1952)
*Navicula granulata* Bailey	*Podochytrium clavatum* Pfitzer	Marine	Intracoastal Waterway at Merrimon, North Carolina	Johnson (1966b)
*Navicula gregaria* Donkin	*Rhizophydium gibbosum* (Zopf) A. Fisch	Marine	Neuse River Bridge, New Bern, North Carolina	Johnson (1966b)
*Navicula mutica* f. *mutica* Kützing [*Navicula mutica* (Kützing) Frenguelli]	*Chytridium braunii* Dang. [*Rhizophydium braunii* Zopf]	Marine	Newport River at entrance to Intercoastal Waterway, Cateret County, North Carolina	Johnson (1966b)
*Navicula oblonga* (Kützing) Kützing	*Chytridium versatile* var. *podochytrioides* Friedmann	Freshwater	Floridsdorfer Wasserpark	Friedmann (1952), Karling (1977)
*Navicula* sp.	*Chytridium perniciosum* Sparrow	Freshwater	Bessemer, New York	Sparrow (1933c), Karling (1977)
	*Chytridium versatile* Scherffel	Freshwater	Mud Pond, McLean, New York	Sparrow (1933b), Karling (1977)
	*Physorhizophidium pachydermum* Scherffel	Freshwater	United States	Sparrow (1960)
	*Rhizophydium clinopus* Scherffel			Scherffel (1930), Sparrow (1960)
	*Rhizophydium gibbosum* (Zopf) A. Fisch	Freshwater	Bessemer, New York	Sparrow (1933c)
	*Rhizophydium globosum* (Braun) Rabenhorst	Freshwater	Ithaca, New York	Sparrow (1933c)
*Navicula* spp.	*Podochytrium clavatum* Pfitzer	Freshwater	Fall Creek and Lloyd Reservation, New York	Sparrow (1933c)
*Navicula viridis* (Nitzsch) Ehrenberg	*Rhizophydium globosum* (Braun) Rabenhorst [*Chytridium globosum* (Braun) Rabenhorst]	Freshwater		Braun (1855, 1856)
*Nitzschia sigmoidea* (Nitzsch) W.Smith	*Chytridium versatile* var. *acaulis* Canter	Freshwater	Bradbourne Park Lake, Sevenoaks, Kent, England; Gaaden, Austria	Canter (1947, 1953), Friedmann (1952)
	*Rhizophydium clinopus* Scherffel			Scherffel (1930), Sparrow (1960)
*Nitzschia* sp.	*Podochytrium emmanuelense* (Sparrow) Sparrow and Paterson [*Rhizidiopsis emmanuelensis* Sparrow]	Freshwater	Cambridge	Sparrow (1933a,a), Canter (1953)
*Pinnularia* sp.	*Podochytrium clavatum* Pfitzer	Freshwater	Germany; Hungary; United States	Pfitzer (1870), Scherffel (1926)
	*Rhizidium braunii* Zopf	Freshwater		Karling (1977)
	*Rhizophydium fusus* (Zopf) Fischer	Freshwater	Hokkaido Prefecture, Japan	Tokunaga (1934)
	*Septocarpus corynephorus* Zopf	Freshwater		Zopf (1888)
*Pinnularia viridis* (Nitzsch) Ehrenberg	*Podochytrium clavatum* Pfitzer	Freshwater	Gaaden, Austria	Friedmann (1952)
	*Podochytrium emmanuelense* (Sparrow) Sparrow and Paterson	Freshwater	Great Britain	Karling (1977)
	*Rhizophydium globosum* (Braun) Rabenhorst			Schroeter (1885)
*Rhizosolenia acuminata* (H.Peragallo) H.Peragallo	*Chytridium* sp. A Johnson, 1967	Brackish	Core Creek, Carteret County, North Carolina	Johnson (1967)
	*Rhizophydium fragilariae* Canter	Brackish	Russell Creek, 2 km north northwest of Beaufort, North Carolina	Johnson (1967)
	*Rhizophydium planktonicum* Canter	Brackish	Flanner’s Beach, North Carolina	Johnson (1967)
	*Rhizophydium* sp.	Brackish	Flanner’s Beach, North Carolina	Johnson (1967)
*Rhizosolenia* sp.	*Rhizophydium planktonicum* Canter	Freshwater	Grand traverse Bay, Lake Michigan	Paterson (1967)
*Stephanodiscus alpinus* Hustedt	*Zygorhizidium* sp.	Freshwater	Schöhsee Lake near Plon, North Germany	Holfeld, (1998, 2000b)
*Stephanodiscus astraea* (Ehrenberg) Grunow	*Podochytrium cornutum* Sparrow	Freshwater	Crose Mere, England	Reynolds (1972)
*Stephanodiscus astraea* var. *minutula* (Kützing) Grunow	cf. *Zygorhizidium planktonicum* Canter	Freshwater		Canter and Lund (1953), Youngman et al. (1976), Holfeld (1998)
	*Zygorhizidium* sp.	Freshwater	English Lake District	Canter and Lund (1953)
*Stephanodiscus hantzschii* Grunow	*Zygorhizidium* sp.	Freshwater		Holfeld (1998)
*Stephanodiscus niagarae* Ehrenberg	*Podochytrium cornutum* Sparrow	Freshwater		Sparrow (1951), Canter and Lund (1953)
*Stephanodiscus parvus* Stoermer and Håkansson	*Rhizophydium horizontale* Paterson	Freshwater	Schöhsee Lake near Plon, North Germany	Holfeld (1998)
*Stephanodiscus* sp.	*Podochytrium lanceolatum* Sparrow	Freshwater		Karling (1977)
	*Rhizophydium horizontale* Paterson	Freshwater	Huron River, Washtenaw County, Michigan, and from Portage Lake, Washtenaw County, Michigan	Paterson (1958b)
*Sundstroemia setigera* (Brightwell) Medlin [*Rhizosolenia setigera* Brightwell]	*Chytridium* sp. B Johnson, 1967	Brackish	Russel Creek, 2 km NNW of Beaufort North Carolina	Johnson (1967)
	*Rhizophydium fragilariae* Canter	Brackish	Beaufort Channel at Duke University Marine Laboratory, Beaufort, North Carolina	Johnson (1967)
	*Zygorhizidium* sp.	Brackish	Adam’s Creek, North Carolina	Johnson (1967)
*Surirella librile* (Ehrenberg) Ehrenberg [*Cymatopleura solea* (Brébisson) W.Smith]	*Chytridium versatile* Scherffel	Freshwater	Hungary	Scherffel (1926)
	*Podochytrium clavatum* Pfitzer	Freshwater	Hungary	Scherffel (1926)
	*Rhizophydium clinopus* Scherffel			Scherffel (1930), Sparrow (1960)
*Surirella ovata* Ehrenberg	*Chytridium surirellae* Friedmann	Freshwater		Friedmann (1953), Karling (1977)
*Surirella* sp.	*Rhizophydium fusus* (Zopf) Fischer	Freshwater	Hokkaido Prefecture, Japan	Tokunaga (1934)
*Synedra* sp.	*Chytridium versatile* Scherffel	Freshwater	brook in St. Andrews, Cambridge	Sparrow (1936a)
	*Rhizidium fusus* Zopf	Freshwater		Zopf (1884)
	*Rhizophydium fusus* (Zopf) Fischer	Freshwater	Sarawak, Borneo	Sparrow (1938)
	*Rhizophydium schroeteri* De Wildeman	Freshwater	Lake Geneva, Switzerland	Pongratz (1966)
	*Rhizophydium* sp.	Freshwater	Grand traverse Bay, Lake Michigan	Paterson (1967)
	*Septolpidium lineare* Sparrow	Freshwater	Chapman’s Garden, Emmanuel College, Cambridge	Sparrow (1933a, 1936a), Canter (1953), Paterson (1958b)
	*Zygophlyctis melosirae* (Canter) Seto [*Zygorhizidium melosirae* Canter]	Freshwater	Douglas Lake, Cheboygan County, Michigan; Grand Traverse Bay, Lake Michigan	Paterson (1958a, 1967)
	*Zygophlyctis planktonica* Doweld [*Zygorhizidium planktonicum* Canter]	Freshwater	Ullvifjarden Lake, Sweden; Douglas Lake, Michigan; Petit Lac, Lac Leman	Canter (1954), Paterson (1956), Pongratz (1966), Karling (1977)
*Synedra* spp.	*Rhizophydium planktonicum* Canter	Freshwater		Rasconi et al., 2012
*Tabellaria fenestrata* (Lyngbye) Kützing	*Chytriomyces tabellariae* (Schroeter) Canter	Freshwater	Bassenthwaite Lake, Buttermere and Crummock Water	Canter (1953), Canter (1951)
*Tabellaria fenestrata var. asterionelloides* Grunow	*Chytridium versatile* Scherffel	Freshwater	Great Britain	Canter and Lund (1953), Canter (1953)
	*Rhizophydium planktonicum* Canter	Freshwater		Canter and Lund (1951b)
*Tabellaria flocculosa* (Roth) Kützing	*Chytriomyces tabellariae* (Schroeter) Canter	Freshwater	Belham Tarn bog near Wray Castle; Floridsdorfer Wasserpark	Canter (1951, 1953), Canter and Lund (1951a), Friedmann (1952)
	*Podochytrium clavatum* Pfitzer	Freshwater	Iburi Subprefecture, Hokkaido Prefecture, Japan	Tokunaga (1934)
	*Rhizophydium* sp.	Freshwater	Grand traverse Bay, Lake Michigan	Paterson (1967)
*Tabellaria flocculosa* (Roth) Kützing [*Tabellaria fenestrata var. intermedia* Grunow]	*Chytriomyces tabellariae* (Schroeter) Canter	Freshwater	Elterwater	Canter (1953)
*Tabellaria* sp.	*Chytridium epithemiae* Nowakowski	Freshwater	Fall Creek in Forest Home, New York	Sparrow (1933b)
	*Chytridium nodulosum* Sparrow	Freshwater	United States	Sparrow (1943¸1960)
	*Chytridium versatile* Scherffel	Freshwater	Surrey	Canter (1953)
*Tabellaria* spp.	*Chytriomyces tabellariae* (Schroeter) Canter	Freshwater		Canter and Lund (1951a, 1953)
	*Rhizophydium* sp.	Freshwater		Canter and Lund (1953)
*Ulnaria acus* (Kützing) Aboal [*Synedra acus* Kützing]	*Zygophlyctis planktonica* Doweld [*Zygorhizidium planktonicum* Canter]	Freshwater	Lake Lanier, Georgia; Esthwaite Water, English Lake District	Karling (1977), Canter et al. (1992), Doggett and Porter (1996), Holfeld (1998, 2000b)
	*Zygorhizidium* sp.	Freshwater	Schöhsee Lake near Plon, North Germany	Holfeld (2000a)
*Ulnaria danica* (Kützing) Compère and Bukhtiyarova [*Synedra ulna var. danica* (Kützing) Grunow]	*Zygophlyctis planktonica* Doweld [*Zygorhizidium planktonicum* Canter]	Freshwater	Lough Creeve, North Ireland	Canter et al., 1992
*Ulnaria delicatissima* (W.Smith) Aboal and P.C.Silva [*Synedra delicatissima* W.Smith]	*Zygophlyctis planktonica* Doweld [*Zygorhizidium planktonicum* Canter]	Freshwater	Blelham Tarn	Canter et al. (1992)
*Ulnaria delicatissima* var. *angustissima* (Grunow) Aboal and P.C.Silva [*Synedra acus var. angustissima* (Grunow) van Heurck]	*Zygophlyctis planktonica* Doweld [*Zygorhizidium planktonicum* Canter]	Freshwater	Switzerland; Italy	Canter and Lund, 1953
*Ulnaria delicatissima* var. *angustissima* (Grunow) Aboal and P.C.Silva [*Synedra delicatissima var. angustissima* Grunow]	*Zygophlyctis planktonica* Doweld [*Zygorhizidium planktonicum* Canter]	Freshwater	Rotsee, Switzerland; Lough Erne, North Ireland	Canter (1967), Canter et al. (1992), Seto et al. (2020)
*Ulnaria* sp. (Kützing) Compère	Rhizophydiales sp.	Freshwater	Lake Inba, Japan	Kagami et al. (2020)
*Ulnaria ulna* (Nitzsch) Compère [*Synedra ulna* (Nitzsch) Ehrenberg]	*Chytridium versatile* Scherffel	Brackish	Flanner’s Beach Station, Neuse River, North Carolina	Johnson (1966c)
	*Septolpidium lineare* Sparrow	Brackish	Flanner’s Beach Station, Neuse River, North Carolina	Johnson (1966c)
*Urosolenia eriensis* (H.L.Smith) Round and R.M.Crawford [*Rhizosolenia eriensis* H.L.Smith]	*Rhizophydium* sp.	Freshwater	Douglas Lake, Cheboygan County, Michigan, and in Portage Lake, Washtenaw County, Michigan	Paterson (1958b)

The table is organized alphabetically by diatom host Accepted Name. Accepted Names were confirmed using AlgaeBase, (Guiry and Guiry, 2023), DiatomBase (Kociolek et al., 2018), and MycoBank. “Marine” and “Brackish” designations for habitat stem directly from the original literature.

### Objective of this review

This review focuses on fungal parasites of diatoms, specifically chytrids (Figures 1A,B). The main undertaking of this work was an attempt to identify all of the first-hand accounts of chytrids parasitizing diatoms (Table 1); additionally, special emphasis is given to what few articles there are describing methods chytrids use to infect diatoms and how diatoms respond to and defend themselves against these parasitic attacks. A summary of the experimental studies exploring diatoms and chytrid parasite interactions is also presented, and chytrid parasites of diatoms in marine environments are also briefly touched on at the end of this review. The latter is a more recent field of study, with much work still needed, especially when compared to freshwater systems (Carney and Lane, 2014; Reñé et al., 2022) though there has been progress in recent years (Gleason et al., 2011; Cleary and Durbin, 2016; Scholz et al., 2016a; Kilias et al., 2020; Buaya et al., 2021a).

## Reported chytrid parasite and diatom host interactions

We reviewed over 200 articles, dating from 1855 to 2023, for firsthand observations or experiments reporting the presence of chytrid parasites on diatoms. Secondhand accounts were used to guide our literature review but are not included as sources. The most notable of these secondhand accounts is Sparrow (1960), though it did have a few firsthand accounts, followed by Karling (1942, 1977) and Canter (1955). The authors consider this review as extensive, but not exhaustive, as some reports or taxa may have been missed, especially among those written in German or Czech in the mid to late 1800s.

From the literature review, we identified a total of 162 named parasitic chytrid-diatom interactions. 63 unique chytrid taxa, in 11 genera, are reported parasitic on 74 unique diatom taxa, in 28 genera (Table 1). 31 of the chytrid taxa parasitized more than 1 host while the remaining 32 were host specific. 31 diatoms were host to multiple chytrid parasites while 43 had 1 specific parasite reported (Table 1).

The most ‘general’ of the chytrid parasites are *Chytridium versatile* Scherffel, *Podochytrium clavatum* Pfitzer, and *Rhizophydium fusus* (Zopf) Fischer, each parasitizing 10 diatom host species. There are eight additional chytrids that parasitize 5 or more hosts (Table 1). Twelve chytrid taxa parasitized only 2 hosts (Table 1). Of these twelve, *Zygophlyctis* aff. *Melosirae* (Canter) Seto, *Rhizophydium gibbosum* (Zopf) A. Fisch, and *Rhizophydium horizontale* Paterson parasitized hosts from the same genus, indicating some hosts may be specific to the generic level. The most extreme example of genus level specificity is *Zygophlyctis planktonica* Doweld, found on 5 taxa within *Ulnaria* (Kützing) Compère, and one taxon of *Synedra* Ehrenberg, though this is likely also an *Ulnaria* that has not been transferred (the other five *Ulnaria* were all originally described as species of *Synedra*) (Williams, 2011). Zooming out, Chytridiales Cohn are the most infectious order with 31 parasites, followed by Rhizophydiales Letcher with 21, Zygophlyctidales K. Seto with 6, and Zygorhizidiales K. Seto with 5 (Table 1). Since 2020, a number of taxa formerly assigned to Zygorhizidiales have been moved to Zygophlyctidales, so these numbers may continue to change with further systematic changes and additional searches (Seto et al., 2020).

On the diatom side, *Melosira varians* Agardh is the most commonly reported host with 15 different taxa parasitizing it. Following *M. varians* are *Asterionella formosa* Hass with 10, *Melosira* sp. with 9, and *Synedra* sp. with 8 parasites, respectively, (Table 1). As a genus, *Melosira* Agardh has been reported to host the most parasites, with 27 parasites on 5 host taxa (Table 1). Twelve diatoms have been reported to host no more than 2 parasites. Of these ten, *Hantzschia amphioxys* (Ehrenberg) Grunow, *Melosira hummii* Hustedt, and *Stephanodiscus astraea* var. *minutula* (Kützing) Grunow are the only ones infected by parasites from one genus. Infected diatoms range the full spectrum of the Bacillariophyta.

The most observed chytrid parasite-diatom host interaction is *Rhizophydium planktonicum* Canter on *Asterionella formosa* with 24 different articles reporting it (Table 1). The second and third most reported combinations are again *Asterionella formosa*, this time parasitized by *Zygophlyctis asterionellae* K. Seto (= *Zygorhizidium planktonicum* Canter) and *Zygorhizidium affluens* Canter with 20 and 14 articles documenting them, respectively (Table 1). The most reported interaction not including *Asterionella formosa* is for the chytrid *Rhizophydium fragilariae* Canter parasitizing the diatom *Fragilaria crotonensis* Kitton, documented on 11 occasions (Table 1). 110 of the 162 total interactions are documented in only one article/report (Table 1). This indicates that while there is wide taxonomic coverage, the data is largely shallow, with only a few interactions containing any repeated observations. The prevalence of studies surrounding *Asterionella* is likely due to its abundance, wide distribution, broad growth conditions, and strong bloom forming abilities (Van Donk and Bruning, 1992; Mekhalfi et al., 2014). *Asterionella* has also been shown to culture well (Canter and Jaworski, 1978; Kagami et al., 2007a; Maier and Peterson, 2014). In addition, initial information gained by studying *Asterionella* may have promoted further study within that system. For these reasons it has been posited as a good model to study diatom-chytrid dynamics. Using *Asterionella* as a “model” diatom host is acceptable when additional specimens/organisms are studied in tandem to discover if any findings are universal; however, this does not appear to be the case for chytrid-diatom interactions, and many findings could be limited to this particular host organism. Further discussion surrounding the experiments conducted with chytrids and diatoms are also expanded upon later in this review.

Of the 162 chytrid parasite-diatom interactions reported, 151 contained information from aquatic habitats. 134 of these interactions are reported from freshwater environments, while only 9 are reported from brackish and 8 from marine (Table 1). All 9 of the brackish reports are from Dr. T. W. Johnson Jr. (two from his 1966c work and seven from his 1967 work). Similarly, seven of the eight marine reports are from Johnson (1966b), while one is from De Wildeman (1893a). The identification of chytrid parasites on diatom hosts is extremely limited for non-freshwater environments, and more research is needed focusing on non-freshwater parasites of diatoms.

Biogeographically, “Hungary” is the most reported locale with 8 interactions, followed by “Grand Traverse Bay, Michigan, United States” with six (Table 1). These reports are not fully indicative of distribution by country, as the reported locality varied by publication, with some as specific as individual bends in a river, and others as broad as countries. Based on the literature, the authors report the United States, the United Kingdom, Germany, Switzerland, and Hungary as the most highly studied. Much of the work in the United Kingdom can be attributed to Canter and collaborators.

DNA surveys have revealed many undescribed novel, ordinal level clades of chytrids (Seto et al., 2016). Additionally, many uncultured chytrids are known, but there has been little success tying molecular data to morphological descriptions, especially at the genus or species level (but see Seto et al. (2023) for recent progress on this front). Modern techniques are expected to continue to reveal novel species and clades, and this table is expected to grow. Future work will be required to tie molecular data to current, or as of yet known, species.

## Methods of infection

### Host penetration

Discussions regarding the specific methods chytrids employ to infect a diatom are rather limited, despite literature on this topic dating back over a 100 years (Canter-Lund and Lund, 1995; Scholz et al., 2017a). Likely one of the first descriptions regarding the method chytrids use to infect diatoms is from Zopf (1888, p 348), who reported that the chytrids *Rhizophydium cyclotellae* Zopf and *Septocarpus corynephorus* Zopf use mycelial tubes to penetrate *Pinnularia* Ehrenberg at the girdle elements (Figures 1E,F), either between the edges of two bands, or directly through the middle of one, but in either case at un-silicified points — thus ignoring the defensive aspect of the siliceous frustule. Subsequent authors reported a similar method of infection (Canter, 1950a; Friedmann, 1952; Canter, 1967; Canter and Jaworski, 1978; Van Donk and Ringelberg, 1983; Ibelings et al., 2004), while Beakes et al. (1992, 1993) went further by reporting that following encystment and germination of a chytrid on *Asterionella,* a germ tube was formed that moved along the frustule and penetrated the host cells by squeezing between the upper and lower girdle lamellae. Scholz et al. (2016a, 2017a), working in marine environments, indicated that, depending on the diatom, the germ tube can punch a hole through the theca, release enzymes to digest an opening, or expand in diameter and wedge a cell open. Each of these three styles was specific to the girdle region of frustules.

In some cases, details are sparser. Canter and Lund (1948) and Canter (1970) mention only that the germ tube enters or penetrates the diatom, with no discussion regarding specific locations on the frustule. Canter (1979), Van Donk (1989), and Van Donk and Bruning (1992) are also limited on details regarding initial penetration, but do include a note that following penetration, an internal rhizoidal system is formed. It is probable that the authors of all of these accounts encountered infections similar to those reported by Zopf, especially considering each of them reported as much in previous or subsequent studies, yet they did lack detail in these specific accounts. A majority of the articles reviewed to build Table 1 did not include any information regarding infection.

There are some accounts that indicate entirely different methods of penetration. Paterson (1958b) reports: “In the developmental sequence of this chytrid, the zoospore encysts on the diatom frustule and sends a penetration tube through the siliceous cell wall of the host. Invasion is not restricted to any particular place on the host cell, although it is possible that entrance may be gained through the areolae” (Figures 1C,D). Canter (1967) while discussing *Zygorhizidium melosirae* Canter on *Melosira* reported the opposite, saying there is no evidence the areolae are used.

Smetacek (1999), while discussing parasites that attack big diatoms, indicates that they target specific sites on the diatom, such as the rimoportulae, and concluded that the frustule provides some protection against parasites. Smetacek (1999) also suggested that certain materials in algal tissues, such as SiO_2_, can restrict the access of parasites, a point that Kagami et al. (2007a) rebuts, citing the “ecologically significant” role that chytrid infections play on diatoms, with the argument for ecological significance being the large number of chytrids infecting diatoms. Smetacek (1999) does not deal directly with chytrids, only broad parasites of diatoms. Kagami et al. (2007a) further discusses how a parasite can infect a diatom, specifying that entry is probable between silicified elements, and proposes *Mallomonas* Perty as an example, which has only one opening not covered by silica, yet is still successfully infected by parasites. Kagami et al. (2007a) does not single out the girdle bands as the sole possible entry point, but instead suggests that any ornamentation on the frustule, provided it is un-silicified, could be exploited. Friedmann (1952), while discussing *Chytridium versatile* var. *podochytrioides* Friedmann infecting *Navicula oblonga* (Kützing) Kützing, also identifies the raphe (Figure 1G), and the connections between cells on *Melosira* species, especially where recent division has occurred, as points of entry (189). Held (1973) indicates that a germ tube may infect a host through any perforation, but this was not specific to diatoms.

**Figure 2:**
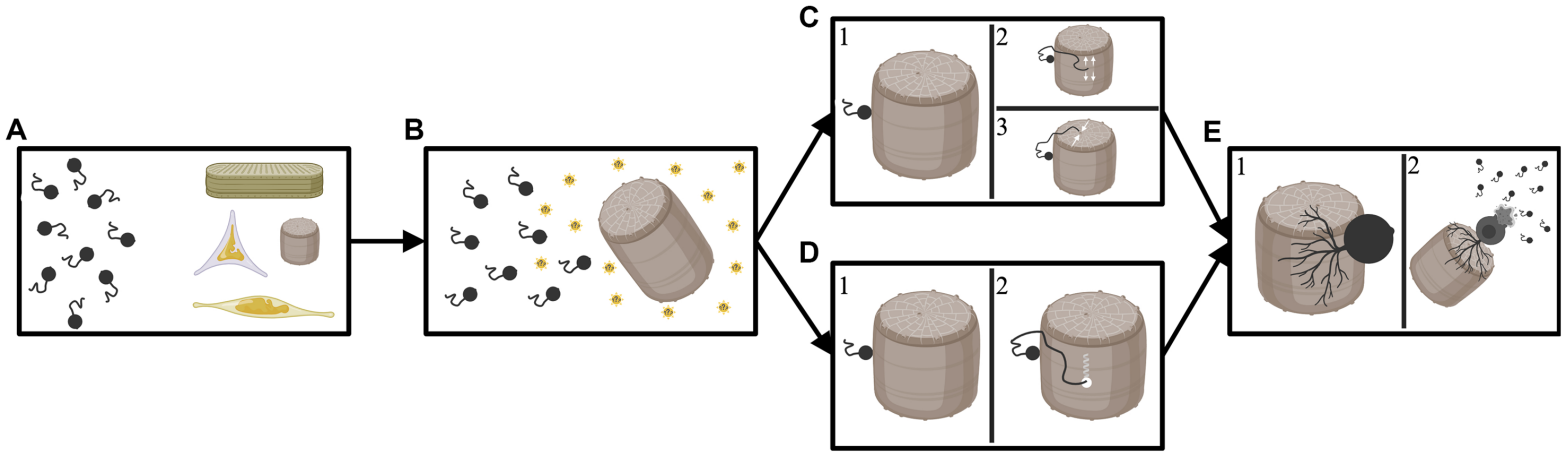
Diagram depicting the potential avenues a chytrid parasite may employ to infect a diatom host. **(A)** Chytrids floating freely with various diatom cells. **(B)** The chytrids are attracted to a specific host that they can encyst upon and penetrate; attraction may be the result of photosynthates released by the diatom (indicated by sunbursts). **(C1)** The chytrid successfully lands and encysts upon the target host. **(C2)** The now encysted chytrid deploys a germ tube which travels along the girdle element of the diatom until it reaches the space between two girdle bands where it can then “wedge” open a space and penetrate the host *or* **(C3)** the now encysted chytrid deploys a germ tube which travels to an opening in the frustule (in this example a pore on the valve face). The germ tube then exploits this opening to enter the diatom host. **(D1)** The chytrid successfully lands and encysts upon the target host. **(D2)** The now encysted chytrid deploys a germ tube which finds a portion of the diatom frustule and then drills through or dissolves the shell. In this scenario the parasite does not utilize any weaknesses or openings in the diatoms silica shell. **(E1)** After entering the diatom frustule the chytrid develops a rhizoidal network. **(E2)** Eventually, the chytrid depletes the nutrients from the diatom host and bursts, releasing new zoospores to infect further hosts. Created by BioRender.com.

These varied reports suggest that chytrids are capable of either finding un-silicified areas of the frustule to exploit, and/or of punching directly through the frustule. At this point, there is not enough evidence to conclude one of the described methods of infections is universal. It is likely that each of the methods occur to some extent, and possibly that additional methods of infection also exist; infection method is inherently difficult to study in these largely uncultured organisms. See Figure 2 for a visual depiction of the potential methods chytrids can utilize to infect a diatom.

As stated earlier, there are relatively few articles that include any mention regarding how infection occurs; among those articles that do, only 7 diatom genera (*Asterionella, Cyclotella* (Kützing) Brébisson*, Licmophora* Agardh*, Melosira, Navicula* Bory*, Pinnularia,* and *Stephanodiscus* Ehrenberg) and 5 genera of chytrid (*Chytridium* A. Braun*, Podochytrium* Pfitzer*, Rhizophydium, Septocarpus* Zopf, and *Zygorhizidium* Löwenthal) have been discussed. This is remarkably low, only 25%, of the 28 diatom genera and 45% of the 11 chytrid genera that are known to have a host–parasite interaction (Table 1). Additional research is necessary to clarify the currently known methods of infection, and to identify and describe as of yet reported ones.

As a final note, almost all of the information discussed above relates to freshwater systems (Kumar, 1980). Wei et al. (2010) reports an almost total lack of knowledge regarding infection of diatoms by chytrids in marine settings. Based on the literature reviewed, the authors of this article agree. Further research is required to determine if the methods, known and unknown, employed in freshwater are utilized in marine settings.

### Host acquisition/attraction

Prior to infection, chytrid parasites must first find and encyst upon a susceptible host. Chytrids rely largely on the movement of water for dispersion, but their ability to swim is highly important once near a target cell (Canter-Lund and Lund, 1995). Some discussion regarding how chytrids orient to and find host diatoms has surrounded the size of the host. Larger diatoms are reported as more susceptible to infection (Holfeld, 1998; Holfeld, 2000a; Kagami et al., 2005, 2007a). Kagami et al. (2007a) described some of the advantages a parasite gains by targeting larger hosts. A short account of several of these advantages is provided here: 1) larger hosts have more resources to offer, allowing for better parasite growth 2) larger hosts require a lower concentration of parasites for successful infection 3) larger hosts are less likely to be grazed, and as a result the lifecycle of the parasite is less likely to be interrupted. There are disadvantages reported for big hosts as well, namely that they may sink more quickly, falling out of the ideal temperature or light range before the chytrid can complete its life cycle (Bruning, 1991b). The role r and k selection (or strategies) play in determining the physical size of diatoms, and the timing of species-specific blooms, has been discussed (Sommer, 1981, 1987), but not specifically in relation to parasites, although there are numerous reports documenting the timing of diatom blooms followed by epidemics (Canter and Lund, 1951b; Canter and Lund, 1953; Van Donk and Ringelberg, 1983; Sommer, 1987; Holfeld, 1998; Holfeld, 2000b). An interesting note on diatom size as it relates to chytrids, following a successful infection, Hanic et al. (2009) reported girdle elements of a host diatom which had expanded to accommodate the parasite’s rhizoidal system.

It can be argued that larger diatoms are easier to find, but in comparison to a body of water, even at microscopic scales, a 2–5 fold increase in volume is miniscule. In bloom scenarios, encountering a host may be trivial for a parasite (Ibelings et al., 2004). The importance of colony formation has also been discussed, where the rhizoids of a parasite may branch to infect multiple cells at once, or discharged zoospores are released in proximity to a new host (Kagami et al., 2007a). A colony is also larger, and thus easier to encounter than a lone cell, but an initial encounter must still occur, and lone cells have been reported to be infected (Table 1). In such cases, signaling has been suggested to explain encounter rates. Held (1973, 1974), discussing mostly *Olpidium* spp., indicated a chemotactic ability helped parasites find hosts; however, in some cases, parasites were still attracted to dead hosts, and could encyst and germinate on them but would not penetrate. Canter and Jaworski (1978) reported dead cells caused no attraction, but could still be encysted upon if incidental contact occurred. Canter and Jaworski (1986) later found that all diatoms attracted parasites, but not all were encysted upon – even non-target hosts could be landed upon. Canter and Jaworski (1981, 1986) also reported that when light was reduced, zoospores would scatter, and when light was returned the zoospores would recongregate around diatoms, suggesting that some product of photosynthesis attracted the parasites. Bruning (1991a) reported similar findings, with almost no infections occurring under light-limited conditions, while light-saturated conditions had extensive infection, and indicated parasites are attracted to hosts *via* chemotaxis. Powell (1994) indicated that chytrids can respond to light, nutrients, pheromones, and host secreted compounds. Holfeld (2000b) reported that chytrids could find hosts at low population densities in addition to high, and further emphasized dead material caused no attraction, but that the presence of non-target diatoms could interfere with attraction, indicating there is something diatoms in general release which attracts chytrid parasites, but the chytrid cannot infect unless it approaches its specific host. While zoospores are attracted to hosts by specific signals, as of yet no single signal has been identified which explains the range of chytrid-diatom interaction. All of these findings support the notion that chemotaxis, or some other form of signaling, occurs which improves the rate at which chytrid parasites encounter diatoms (Bruning, 1991a; Scholz et al., 2016a, 2017a). It is not currently clear if chytrid parasites are only attracted to diatom hosts that they can actually infect, or if all diatoms attract chytrid parasites, and then the parasites are only capable of causing an infection on specific hosts. Frenken et al. (2017) indicated chemical interactions at the host’s surface as a likely mediator of host–parasite recognition. More work is required to determine host parasite recognition and infection, especially how this varies across different parasites and their hosts.

### Host specificity

A number of authors have looked into host specificity between chytrids and diatoms (De Bruin et al., 2004; Ibelings et al., 2004; Gleason et al., 2011; Seto et al., 2020). The consensus has been that some chytrids are highly host specific, but no comments exist for many other reported interactions. Based on Table 1, this does seem to be the case, as many parasites have been reported on only one host, while others (*Chytridium versatile* as an example) have been found on multiple diatoms across many genera. As discussed earlier, no universal method of infection is known for chytrid-diatom interactions, and it is likely that many, including as of yet described methods, exist. Interestingly, at least two accounts exist documenting chytrid zoospores that have been attracted to, and attached to (although rarely), a non-target diatom host (Held, 1974; Canter and Jaworski, 1981). In both cases, the zoospore died and was unable to successfully infect the host (Canter and Jaworski, 1981). Held (1974) describes this as “attractive” but not “attachable,” though he may have been discussing one diatom *Tabellaria* Ehrenberg ex Kützing, and not non-target hosts as a whole. Investigations looking into unique or varied ornamentation on diatom frustules, paired with specific germ tube morphology may help explain this “attractive” but not “attachable” phenomenon.

Perhaps chytrid parasites and their diatom hosts have co-evolved, or an evolutionary arms race has occurred, and as a result chytrids parasites are only able to infect their target host. Frenken et al. (2017) mention that only certain strains of a host within the same species can be successfully infected and parasitized and indicate host surface traits as possible explanations for resistance, but this is not specific to diatoms. Much research is required before any claims regarding coevolution, or an arms race, between chytrid parasites and host diatoms can be made. Ibelings et al. (2004) discusses host–parasite coevolution from a genotypic standpoint, indicating that the conditions for coevolution exist, but conclusive evidence is lacking. Furthermore, they identify that a few diatom cells, sunken out of the infective zone, may serve as a refuge for hosts to replenish populations, and thus diatoms may not need to adapt to counter the parasite. A much more expansive discussion of coevolution involving chytrids in general, and the next required research steps, can be found in Frenken et al. (2017).

Aside from phenotypic features, De Bruin et al. (2004) showed that differences in genotype may limit parasite effectiveness. Among studied colonies of *Asterionella formosa* in Lake Maarseveen, they found that different genetic makeups within species of diatom were more, or less, susceptible to infection by *Zyorhizidium planktonicum*. Likewise, using molecular methods, Seto et al. (2020) found that *Zyorhizidium planktonicum* actually represented two different species (*Zygophlyctis asterionellae* and *Z. planktonica*), parasites specific to *Asterionella* and *Synedra,* respectively. And Gsell et al. (2013a) indicates that high levels of parasitism actually increase genetic variation among *Asterionella formosa* colonies, possibly due to suppressing one clonal population from dominating the environment. Frenken et al. (2017) indicate morphological resolution as a knowledge limiter, as current methods lack the ability to reliably place individuals at the species level, which may in turn mask specialists from generalists. Further investigation may help elucidate additional scenarios where multiple species are lumped, further clarifying the prevalence of generalists and specialists among chytrid parasites.

## Diatom defense mechanisms

How diatom hosts survive, despite epidemics that can cause a near 90% mortality rate year after year, is a recurring question in reviews covering chytrid-diatom interactions (De Bruin et al., 2004; Ibelings et al., 2004; Kagami et al., 2007a). A few mechanisms have been suggested to answer this question, including: the Red Queen Hypothesis, hypersensitivity responses, “fungicide” production, deep water seed banks, and one account of developed immunity.

The Red Queen Hypothesis is one of the more commonly discussed defenses; it posits diatoms evolved to sexually reproduce in order to expand genetic diversity and fight chytrid parasitic infection (De Bruin et al., 2004; Ibelings et al., 2004; Kagami et al., 2007a). The account from De Bruin et al. (2004) is included here, as they investigated this in some detail with the diatom *Asterionella formosa* and the chytrid parasite *Zygorhizidium planktonicum*, and found an “unexpected” level of genetic variation among the diatom population, agreeing with the hypothesis that genetic variation slows parasitic attacks. However; they failed to identify auxospore formation, a key indicator of sexual reproduction in diatoms, though it is assumed to occur based on observed sexual reproduction in other diatoms, and the genetic diversity among populations of *A. formosa*, just at slow intervals. Jephcott et al. (2017) provides a more recent discussion of the Red Queen hypothesis, as well as rebuttals, and the relation of Cheshire Cat dynamics, in host diatoms and their chytrid parasites.

Hypersensitivity responses in diatoms are less well reported, with Canter and Jaworski (1979, 1982) being among the only to document it in *Rhizophydium planktonicum* on *Asterionella formosa* and *Rhizophydium fragilariae* on *Fragilaria crotonensis*. Additional discussions surrounding this response in diatoms following chytrid infection can be found in Canter-Lund and Lund (1995), Ibelings et al. (2004), Kagami et al. (2007a), and Scholz et al. (2016a). A hypersensitivity response results in the almost immediate death of the host (*via* programmed cell death) after infection by the parasite (White et al., 2000). This fast response cuts the life cycle of the parasite short, preventing it from forming a sporangium and multiplying, thus limiting the infectious potential of parasite populations as a whole (Canter-Lund and Lund, 1995; Ibelings et al., 2004). Most of the information surrounding hypersensitivity responses stems from work done on terrestrial plants (Kagami et al., 2007a). Canter-Lund and Lund (1995) indicate that this response is limited in *Asterionella formosa* to only *Rhizophydium planktonicum*, and not any other parasite, and Ibelings et al. (2004) reported it as strain specific, not species specific. Further work could clarify how widespread this phenomenon is among different diatoms and why certain strains elicit this response and others apparently do not.

Information regarding “fungicide” production in diatoms stems from Pohnert (2000); they indicated that mechanically wounded diatoms produced fatty acid derived metabolites which resulted in the release of a,b,g,d-unsaturated aldehydes; these released products were then shown to act as fungicides against *Schizophyllum* Fries and *Aspergillus P. micheli* ex Haller (Ibelings et al., 2004), but this antifungal activity has not been examined specifically for chytridiaceous fungi (Kagami et al., 2007a), and further work is required. Ibelings et al. (2004), Kagami et al. (2007a), and Scholz et al. (2016a, 2017a) all discuss this response in some detail.

Deep water seed banks refers to the scenario where some diatom resting spores sink to the sediment, putting them below the range of survivability for chytrids, and thus allowing them to dodge the parasitic epidemic (Sicko-Goad et al., 1989; Ibelings et al., 2004; Kagami et al., 2007a). At the subsequent water column turnover, these diatoms are resuspended, where they reseed the previously decimated population (Sicko-Goad et al., 1989). As the course of the year carries on, some diatoms are consistently transitioning in and out of the resting spore stage and sinking to the sediment or being resuspended (Sicko-Goad et al., 1989). Those that remain in the water column are parasitized and killed off during epidemics. This results in a cycle that can explain how diatoms are able to replenish their stock despite such massive losses. The basis of deep water seed banks as a defense mechanism in diatoms stems from Bruning (1991a,b,c,d), who showed that at low temperature and light, *Asterionella* could grow, but the chytrid could not. There are additional studies also reporting chytrids detaching from diatom hosts when removed from light (Canter and Jaworski, 1981; Canter and Jaworski, 1986). Experimental work is likely needed to determine how seed banks may serve as refugia from parasitic chytrids.

We did find one account of a diatom developing “immunity” to a parasite following an initial infection in Friedmann (1952). He reports cultures of *Melosira varians*, which had survived infection by *Rhizophydium melosirae* Friedmann and *Podochytrium clavatum* could not be later reinfected with fresh parasites, despite a remaining healthy abundance of diatom cells. Friedmann (1952) does not describe the approach he took to re-expose surviving cells to the fungal parasites, nor does he mention this finding anywhere else. Likewise, no subsequent experimenter or reviewer reported immunity following infection. Further work is required to determine if any diatom cells develop immunity.

It is possible that some combination of these factors, and potentially as of yet known ones, may contribute to diatom defense efforts. No studies have been carried out looking at any two or more factors together. Future work should focus on identifying new factors while also refining what is currently known about existing ones.

## Factors affecting the infection of diatoms by chytrids

A number of experiments investigating the infection of diatoms by chytrid parasites have been published. These include initial forays to determine what, if any, impact chytrids have on host diatom populations. Later experiments examined the impact biotic and abiotic factors have on parasitic interactions. Here we summarize a few experimental methods, some observational reports, and the findings of various experiments regarding light, temperature, nutrients, host specificity and grazing on chytrid-parasite diatom-host interactions.

### Culturing

Most experiments dealing with diatoms and chytrid parasites rely on some level of culturing. Canter and Lund (1948) were among the first to attempt such culturing using *Rhizophidium*, but they failed, though they did mention some contemporaries, namely Friedmann, had reported success. Friedmann (1952) managed to grow fungal parasites on benthic diatoms in enrichment cultures, but not on any diatoms from the plankton. One of the first described, and replicable methods of culturing stems from Canter and Jaworski (1978), who established co-cultures of *Rhizophydium planktonicum* and *Asterionella formosa*. Canter and Jaworski (1978) contains a well described method for those looking to establish their own cultures. More recently, Kagami et al. (2007a), Maier and Peterson (2014), Seto et al. (2016), and Seto et al. (2020) have described methods for reliably establishing and studying cultures of diatom hosts and chytrid parasites from environmental samples.

### Light

An early record of the role light plays in the parasitic interaction between chytrids and diatoms stems from Friedmann (1952) who reported that a loss of light caused *Rhizophydium melosirae* individuals to release from their *Melosira* hosts. Later observations from Canter and Jaworski (1979, 1980) indicated that the chytrid *Rhizophydium planktonicum’s* motility was impacted by light intensity, with erratic or no movement occurring in darkness.

A formal assessment and experiment regarding the role of light followed shortly thereafter in Canter and Jaworski (1981). In the 1981 study, Canter and Jaworski used clones of *Asterionella* and *Rhizophydium planktonicum* to test a number of different hypotheses, among which included the reactions *R. planktonicum* had to *A. formosa* under light, dark and alternating conditions. To summarize their findings, under light conditions, zoospores moved in a free flowing manner until they neared a host (often many zoospores would surround a single host cell, or colony of cells, at once), at which time their movements became erratic. Following the period of erratic motion, zoospores of the chytrid would sometimes “freeze” where contact between the parasite and the host would occur. The zoospores of the parasite, often many on one cell, were able to infect the host, and a normal life cycle played out. Under dark conditions, zoospores never displayed the erratic movement, did not congregate around cells, and did not encyst upon hosts. When moving the chytrid from light to dark, zoospores would disperse away from the diatom host and only return once light had been restored. Canter and Jaworski (1981) also indicated that a full removal of light is not needed to lower the infection rate, and that just reducing the light intensity dramatically reduced successful encystments, and that this change in “attraction” could be seen in as little as 30 s. They also reported that when moving a chytrid from dark to light it was immediately able to find and orient to light incubated *Asterionella* hosts, suggesting the chytrid is likely attracted to a photosynthetic compound released by the host, a point further reinforced in Canter and Jaworski (1986). In contrast, light incubated zoospores added to an inoculum of dark incubated diatoms took a few moments to orient and find hosts. This delay is likely due to a lag between when the diatom host is first exposed to light and when photosynthesis actually starts. Sicko-Goad et al. (1989) report a similar lag in diatom photosynthetic production for rejuvenated resting spores across different temperatures. Around the same time as Canter and Jaworski (1981), and a little before, a number of other researchers reported similar findings regarding host–parasite interactions and light, but these were for either non-chytrids, or non-diatoms (Kumar, 1978).

Bruning released a series of articles discussing the roles of abiotic factors on chytrid parasitism of diatoms in 1991, the first two of which centered on light. Under light-limited conditions, Bruning (1991a) found that parasites developed fewer zoospores–though they developed at the same time rate. Additionally, Bruning (1991a) found that at light limited conditions, *Asterionella* hosts were less susceptible to infection, and below a minimum intensity threshold no infection occurred, though there was still enough light for the diatom to grow and reproduce. Growth under low light conditions is suggested as part of the method *Asterionella* populations exploit to explode in population during the early season (when ice still covers the water), and the subsequent increase in light allows the chytrid to catch up and eventually overtake the host, causing an epidemic (Van Donk and Ringelberg, 1983; Bruning, 1991a). Though late season light limiting conditions do help cause epidemics as they reduce the hosts growth; epidemics can still occur during high growth phases of both parasite and host (Bruning, 1991b). Temperature also plays a role in this interaction, with different light and temperature regimes leading to higher or lower infectivity, this is discussed more in the following section (Bruning, 1991d). The third article of the series (Bruning, 1991c) covered phosphorus limitation, and is discussed in a lower section of this work. All of Bruning’s (1991a,b,c,d) studied *R. planktonicum* on *Asterionella.*

All of the experiments dealing with light were limited to just two diatom host genera, *Melosira* and *Asterionella,* and one genus of chytrid parasite, *Rhizophydium*. Due to the limited number of examined partners, it is difficult to draw conclusions for all diatom and chytrid interactions, and future work needs to be performed to expand on these findings. We recommend *Fragilaria* Lyngbye*, Tabellaria, Navicula, Synedra, Stephanodiscus* and *Pinnularia* as diatom genera to study, as they are known hosts to many chytrid parasites and have been successfully cultured in the past (Canter and Jaworski, 1983; UTEX Culture Collection of Algae, 2023). Similarly, we advise representatives from *Chytridium* and new representatives of *Rhizophydium* be examined, as they parasitize many diatoms. Representatives from *Zygorhizidium* are also likely to shed new light in this area, but to date these have been difficult to culture. All of the data so far is for freshwater environments, and studies examining marine environments are needed; there are already some groups pushing this work forward (See Marine section for more information).

### Temperature

Similar to light, initial experiments examining the impact of temperature on chytrid-diatom parasitic interactions stemmed from observations made from field samples. Among the first true experiments to explore the role of temperature comes from Van Donk and Ringelberg (1983). In this work, the authors tested the growth of the diatom *Asterionella formosa* in conjunction with the chytrid *Zygorhizidium planktonicum* at 4 different temperatures: 1.5°C, 5°C, 10°C, and 18°C. At 1.5°C, *Z. planktonicum* did not multiply, nor did it display any parasitic tendencies (Van Donk and Ringelberg, 1983). In contrast, *A. formosa* grew well. At the higher temperatures, *Z. planktonicum* successfully parasitized *A. formosa*. Combined with observational data it was concluded that above 4°C, chytrids could cause epidemics, but below this temperature the diatom would thrive and remain uninfected (Van Donk and Ringelberg, 1983).

Similar to the work of Van Donk and Ringelberg (1983), Bruning (1991d) also examined *Asterionella formosa*, but this time with the parasite *Rhizophidium planktonicum*. Bruning (1991d) found that at low temperatures (*circa* 2°C), *R. planktonicum* zoospores took longer to reach maturity, and had a shorter window of infectivity. As temperatures increased, the development time got substantially shorter, and the window of infectivity grew longer. Bruning (1991d) also assessed the likelihood of epidemics at different temperatures, and reports that above 7°C light and temperature intensities play less of a role, but below 5°C epidemics can only occur if the diatom host is subjected to limited light, and a resulting impeded growth rate. Kudoh and Takahashi (1990), also examining parasitic fungi on *Asterionella formosa*, reported similar results from observational data and experiments they carried out in a shallow eutrophic lake, with *Asterionella* dying due to fungal infection less likely at cold temperatures, but increasing with temperatures. Additionally, Kudoh and Takahashi (1990) also indicated that at temperatures below 5°C, while not impossible, mortality due to fungal infection of the diatom host was “severely inhibited.”

A generalization of the impact temperature has on the chytrid parasite diatom host interaction is presented in Kudoh and Takahashi (1992). To briefly summarize: 1) at low temperatures (near 0°C) both the diatom and chytrid have inhibited growth rates 2) the diatom is able to start growing earlier as water temperature begins to rise, allowing the population to race ahead of the chytrid 3) as temperatures continue to rise, the chytrid enters a growth phase 4) eventually the chytrid outpaces the diatom, and an epidemic occurs, until finally 5) the diatom population collapses. It is also suggested that once the diatom reaches a sufficiently low population density, the encounter rate between the host and the parasite becomes too low to maintain a parasitic population, and the diatom is free to grow again (Kudoh and Takahashi, 1992).

More recently, a number of results have been reported for marine and freshwater systems (Wei et al., 2010; Gsell et al., 2012, 2013a,c). The marine report does not focus specifically on diatom and chytrid interactions, but does include both independently (Wei et al., 2010). It is not clear in these systems exactly what role temperature plays, as there are conflicting reports that parasites become active only at higher temperatures (~30°C), or the parasite dies above ~16°C (Wei et al., 2010). The report from marine environments also indicates isolates from different regions have different suitable temperature ranges, so one conclusion may not be broadly applicable. The other studies (Gsell et al., 2012, 2013a,c) examined how different genetic strains of *Asterionella formosa* reacted to a parasite across a broad temperature range, and found that each strain had a range where it was more likely to be infected, and extreme bounds that served as growth refuges that the chytrid could not parasitize. Their results also indicated that no one strain was better across all temperatures, but instead each thrived or struggled accordingly (Gsell et al., 2012¸ 2013c). The resistance to parasitic infection at different temperatures that these strains display is suggested as a possible maintainer of diversity among *Asterionella*.

Studies examining the impact of temperature share many of the limitations as those examining light. Namely, only one species of host has been studied, *Asterionella formosa*, and only two genera of parasites, *Rhizophydium* and *Zygorhizidium*. Additional work, expanding the information on hosts and parasites is required before large generalizations can be made. This work largely surrounded freshwater ecosystems as well, and studies in marine environs will be needed. Wei et al. (2010) does provide a start, though it does not include chytrids on diatoms.

### Nutrients

Only two studies were found that document the role nutrients, and nutrient limitation, play in the interactions between diatoms and chytrid parasites, with phosphorus being the only nutrient discussed (Bruning and Ringelberg, 1987; Bruning, 1991c). At normal conditions, sporangia took on average 45 h to develop, and produced around 26 zoospores; when the host was phosphorus limited, sporangium development decreased to around 40 h, and the number of zoospores produced also decreased, to an average of 9 (Bruning and Ringelberg, 1987). On the diatom host side, parasitic infections occurred at a higher rate when phosphorus was not limited, and when it was limited hosts were less susceptible to infection (Bruning, 1991c). Despite this, at phosphorus limited conditions, diatoms are up to 2.5 times more susceptible to epidemic infections, as the parasite is able to outgrow the diatom host (Bruning and Ringelberg, 1987; Bruning, 1991c). Similar to light and temperature, the diatom host examined in these studies was *Asterionella formosa* and the chytrid parasite was *Rhizophidium planktonicum* (Bruning and Ringelberg, 1987; Bruning, 1991c).

Gleason et al. (2008) and Gleason et al. (2011) examined the effects that other abiotic and factors such as salinity have on the growth of fungi, but this is not specific to chytrids, nor is it in relation to chytrids parasitizing diatoms. These reviews may serve as jumping off points for future work. Gleason et al. (2008) was specific to freshwater systems while Gleason et al. (2011) looked in marine environments. It should be noted that Gleason et al. (2011) focused on fungal parasites and algae in general, and was not specific to chytrids and diatoms, with the one reported diatom-chytrid interaction stemming from a report by Hanic et al. (2009), and that report did not provide species identifications.

### Host specificity and recognition

Host specificity has also been reported observationally, though few studies exist experimentally testing it. Canter and Jaworski (1978, 1982) and Canter et al. (1992) carried out much of this experimental work surrounding how chytrid parasites identify their host diatom. Canter and Jaworski (1978) examining the infectivity of *Rhizophydium planktonicum*, found all of their clones of *Asterionella formosa* were susceptible to infection by *t*he parasite, with some clones more violently parasitized, and very few clones bearing dead zoospores, which indicated some individuals are resistant/survive an initial infection. They also attempted to infect other diatoms, species of *Tabellaria*, *Cyclotella*, *Synedra*, and *Fragilaria*, with *R. planktonicum* cultures pulled from *A. formosa*. Neither *Tabellaria* or *Cyclotella* were successfully infected, though they did note that on rare occasion a zoospore would encyst, but never grew. *Synedra* and *Fragilaria* were infected, but it was exceedingly rare, as the initial encystment was unlikely, and of the zoospores that did manage to encyst, few grew. In all cases, epidemics did not occur except on the initial *Asterionella*. Canter and Jaworski (1978) also examined infection on dead and dying *Asterionella* cells. To do this they killed the diatom using heat. Even after death, these cells were encysted upon, but no growth of the parasite occurred due to a lack of exploitable organic material. Other materials tested had no encystment or growth. From their results, Canter and Jaworski (1978) concluded that highly susceptible strains of *Asterionella* exist, though more study would likely reveal immune or resistant strains. They also revealed that, while possible to cross infect in experimental circumstances, *R. planktonicum* was most successful at infecting *Asterionella*, its natural host.

Expanding on their 1978 work, Canter and Jaworski (1982) performed an experiment investigating the infectivity of *Rhizophydium fragilariae* on two distinct strains of *Fragilaria crotonensis*. They found that one clone was highly susceptible to infection, while the other had negligible infection. When tested with populations from a different source, the findings remained the same, with the susceptible host again being highly infected and the other host having few to no parasites on it. In contrast, a second, lesser known parasite of *F. crotonensis* was examined, and it successfully infected the *F. crotonensis* clone not parasitized by *R. fragilariae,* while the host susceptible to *R. fragilariae* was not infected by this new chytrid. From these results, Canter and Jaworski (1982) concluded that resistant and susceptible strains of the same species exist, and in addition to some generalists, there exist parasites that are specific to their host.

Canter et al. (1992) similarly looked at infectivity, this time using the parasite *Zygorhizidium planktonicum*, which could infect *Synedra* and *Asterionella*. Specifically, they reported that some clones of *Z. planktonicum* could only infect individuals of *Synedra*, while other clones could only infect individuals of *Asterionella*. *Zygorhizidium planktonicum* clones were still drawn to the diatom host they could not infect, which left the authors wondering if a mechanical issue prevented cross infection. Seto et al. (2020) separated the two clones of *Z. planktonicum* into two new species in a new genus, *Zygophlyctis asterionellae* and *Zygophlyctis planktonica*, with *Z. asterionellae* only reported to infect *Asterionella*, while *Z. planktonica* has only been reported to infect *Synedra*. Canter et al. (1992), and the new descriptions from Seto et al. (2020) further emphasized that some chytrids could only successfully infect and complete their life cycles on specific hosts.

Additional experiments and studies looking at host specificity have also occurred, though not to the same extent as those listed above. Maier and Peterson (2014) found that chytrids parasites of *Asterionella formosa* from the Columbia River were unable to cross infect individuals of *Tabellaria*, *Fragilaria, Aulacoseira* Thwaites, and *Synedra* under experimental conditions. In contrast, of 8 chytrid strains, Kagami et al. (2020) identified, 5 (all isolated from *Aulacoseira granulata* (Ehrenberg) Simonsen) could successfully infect 4 different species of diatom (*Aulacoseira granulata, Aulacoseira ambigua* (Grunow) Simonsen, *Ulnaria* sp., and *Asterionella formosa*), while 3 strains could only infect their natural host. Taken together, these studies indicate there are both generalist and specialist interactions occurring, even down to the species level.

### Effects of grazing on chytrid-diatom interactions

Much of the work between grazing and the diatom chytrid dynamic stems from initial explorations of the mycoloop. Large diatom cells, e.g., *Asterionella formosa*, are less susceptible to zooplankton grazing, as they are outside the particle range of common grazers like *Daphnia* Müller (Kagami et al., 2005). Kagami et al. (2005) tested two hypotheses relating to diatom-chytrid dynamics and grazers. The first hypothesis was that parasitism by *Zygorhizidium planktonicum* or *Rhizophydium planktonicum* makes *Asterionella formosa* more susceptible to grazing, as the parasites weaken cell to cell connections and break up colonies, reducing the overall size of the diatom. The second hypothesis is that chytrid parasitism makes *A. formosa* less susceptible to grazing, as parasitism had been reported to cause colonies to group up, increasing the overall size of the diatom. To test this, they placed uninfected cells of *A. formosa* into 6 flasks, and infected cells into 6 flasks; they then added *Daphnia* taxa to 3 of each of the 6 and let the populations go. They found that “fungal infection did not make *A. formosa* more vulnerable to *Daphnia* grazing. Fungal infection did, however, induce aggregation of colonies, which seemed to make *A. formosa* somewhat less susceptible to *Daphnia* grazing” (Kagami et al., 2005).

Building on this study, Kagami et al. (2007b) performed experiments to examine if the presence of *A. formosa* and *Z. planktonicum* together in a flask resulted in more *Daphnia* biomass than *A. formosa* individually or *Z. planktonicum* individually. They found that fungal parasites did support growth of *Daphnia.* Upon successful encystment, the parasite would complete its life cycle, part of which entails the creation and release of new zoospores. These zoospores could then be grazed on by *Daphnia*, thus shifting the material which had otherwise been trapped in the diatom to the grazer *via* the parasite. Kagami et al. (2007b) used populations that were at 60% infection, for epidemics reaching the upper reported infection ranges (90% or more) the transfer from diatom to grazer likely increases, and the zoospores are likely an important food source for zooplankton grazers. Using models, Miki et al. (2011) also showed a similar occurrence to be happening, where a fungal parasite was aiding in material transfer from inedible diatoms to grazers. Miki et al. (2011) did report that this can become inverted, with less material being transferred, and indicated that high fungal growth rate and rich host tissues are needed for a positive transfer to occur. The mycoloop is now understood to be a component of food web cycling in freshwater habitats (Kagami et al., 2014), less is known of this system in marine settings.

Lehman and Sandgren (1985) discuss the impacts grazers have on diatoms in some detail, though this is not in relation to chytrid parasitism. They indicate that grazing can cause the populations of some larger, colonial diatoms to increase, but nutrient limitation sets a cap to this growth. Increased grazing, which selects for large, ungrazeable diatoms, is likely to lead to higher rates of parasitism as chytrids better parasitize these diatoms (Maier and Peterson, 2014), which they then break into smaller organic matter which can in turn be grazed constituting the mycoloop. Additionally, larger diatom individuals have been reported to increase the fecundity of their chytrid parasite (Holfeld, 2000a).

### Turbulence/disturbance

We encountered two studies that have examined the impact of turbulence on the interactions between chytrid parasites and diatom hosts (Bertrand et al., 2004; Maier and Peterson, 2014). The first, Bertrand et al. (2004), sampled *Asterionella formosa* from nine reservoirs on the Durance and Verdon Rivers, each with a different suite of hydrodynamic characteristics. Bertrand et al. (2004) looked at chytrids in general as parasites, and did not identify any to the genus level. For the experiment, disturbance was defined as “any discrete events which disorganize the structure of an ecosystem, community or population and affect the food supplies, substrate availability or physical environment.” Bertrand et al. (2004) identified 3 groups of dams: 1) those with deep water, a long retention time, and “low” disturbance 2) those with intermediate depth, an intermediate retention time, and intermediate disturbance 3) those with shallow water, a short retention time, and “high” disturbance. *Asterionella formosa* cells from the intermediate disturbance group were found to be more parasitized by chytrids. Bertrand et al. (2004) point out that host cell densities were higher among the intermediate disturbance group, and this likely contributed to the higher rate of parasitism. Among the high disturbance group, both the chytrid and the diatom were negatively affected, with the chytrid’s ability to swim impeded, and the diatom’s ability to grow and form colonies reduced, lowering the population density and decreasing the rate of infection. In the low disturbance habitats the chytrid is not adversely affected, but no mention of *Asterionella* was made; we propose that an increased sinking rate due to lack of disturbance could cause the host cells to fall out of the infectious zone faster, reducing parasitism. Reñé et al. (2022), do report Chytridiomycota corresponding to diatoms in the top 20 meters of water, though this is in a marine system. Across all disturbance levels, colonial *Asterionella* was more likely to be infected than lone cells.

The second study examining the effect of turbulence on diatoms and chytrids is from Maier and Peterson (2014), and, in regards to turbulence, was mostly observational on *Asterionella formosa*, though experiments further testing host specificity are included. The report indicates that the damming of rivers or other lotic systems “greens” waterways, and increases the rates of parasitism as diatoms are better able to grow in the phytoplankton due to less disturbance, and that chytrids subsequently infect the larger populations. The authors indicate that further work is needed to determine what role turbidity and damming will have on chytrid diatom interactions in the Columbia River.

### Notes and issues

As can be seen from these studies, much of the experimental work surrounding chytrid parasites and diatom host interactions is based on *Asterionella.* This is also indicated in the chapter discussing the ecology of chytrid and aphelid parasites of phytoplankton by Jephcott et al. (2017). While the depth of some of these studies are extensive, the narrow breadth makes it hard to draw broad conclusions, as these reported interactions may be specific to *Asterionella*, and not indicative of all, or even most, diatom hosts. Future work involving other diatom hosts is required to flesh out this field. Frenken et al. (2017) point out difficulties related to culturing both diatoms and chytrids as barriers moving forward; they additionally acknowledge a lack of interest in the field in the past. For the future, they suggest the application of automated single cell sorting using flow cytometry as a potential aid in both culturing and taxonomy, though difficulties can arise in identifying organisms using these methods, especially to the species or genus level. Collaborative work between phycologists, specifically diatomists, and mycologists focusing on zoosporic fungi may be beneficial in identifying novel host–parasite interactions.

## Diatom chytrid interactions in marine environments

As a final section, we discuss and review the literature surrounding chytrid parasites and diatom hosts in marine ecosystems. Overall, this is a much less studied field than freshwater systems, with much of the work occurring in the last 15 years (Gleason et al., 2011; Cleary and Durbin, 2016; Scholz et al., 2016a; Kilias et al., 2020; Buaya et al., 2021a,b). The field has also skewed towards Oomycete parasites over chytrid parasites, but there do exist accounts of both. Future work should continue to emphasize this area, as the interactions between chytrids and diatoms in the open ocean is likely massive, especially taking into account the mycoloop (Kagami et al., 2007b), the oxygen production of diatoms, and the role diatoms play in carbon (Smetacek, 1999; Smetacek, 2018) and silica sequestration (Garvetto et al., 2019). Many diatoms used for biofuel are also marine, so there exists the risk of major economic impacts from marine parasitic chytrids.

Sparrow (1936b) is among the first to document fungi infecting diatoms in marine environments, though the fungi were from the Oomycetes, not the chytrids, *Ectrogella perforans* on *Fragilaria unipunctata* Lyngbye and *Licmophora abbreviata* Agardh. Sparrow (1936b) did identify *Rhizophydium globosum* (Braun) Rabenhorst in his marine samples, a chytrid reported to parasitize numerous diatoms in freshwater systems (Table 1), but he did not indicate any parasitized diatoms at the time of his sampling. Perhaps most importantly from this work, Sparrow observed fungi in marine samples, including chytrids, and indicated that future study would almost certainly expand known taxa lists.

In contrast, Kohlmeyer and Kohlmeyer (1979) posit that oceans, despite their size, likely have drastically fewer fungal species than freshwater or terrestrial environments, ~1% of the total species count. Van Donk and Bruning (1992) also indicate that phycomycetes occur to a lesser degree in marine environments, and Richards et al. (2015) points out that currently less than 1% of fungi species are reported from marine habitats. Part of Kohlmeyer & Kohlmeyer’s (1979) argument stems from the stability of ocean systems, with narrower temperature and salinity gradients. Additionally, even though the oceans are massive, covering ~70% of the Earth’s surface, Kohlmeyer & Kohlmeyer (1979) indicate that a majority of the fungi live near to shore, where organic substrates are in abundance, and the ocean center is a desert for all but those fungi growing on planktonic animals. A detailed discussion of the diversity on land vs. in the oceans is beyond the scope of this work and exists elsewhere (see May, 1994 for a start). One note from May’s (1994) work is included: the number of taxonomists working in ocean systems is drastically lower than for land or freshwater systems, and this could partially explain the difference in species numbers in marine settings. As pointed out earlier, there have not been as many taxonomists working on chytrids and diatoms in conjunction in freshwater systems, so marine habitats are likely even more ignored. Kohlmeyer & Kohlmeyer (1979) also indicate algae-inhabiting fungi are less studied than fungi living on substrates such as wood as they are harder to find, culture and identify, and they posit algae-inhabiting fungi are rare, and limited in distribution, though both historic and recent work indicates this is not the case, and algae-inhabiting fungi are likely present in the whole ocean, just understudied (Sparrow, 1936b; Richards et al., 2015).

Richards et al. (2015) point out a “significant number of chytrid-like lineages” found from environmental DNA based methods that have not been described. Kohlmeyer and Kohlmeyer (1979) identified 32 “higher fungi” that are parasitic in marine environments. Not one of these 32 is from Chytridiomycota, and no diatoms were examined as hosts. Kohlmeyer & Kohlmeyer (1979) seem to have missed the work of Johnson (1966a,b,c, 1967), though his work was in brackish and saline waters, and may not have met their criteria for “marine.” Johnson (1966a,b,c, 1967) identified 16 chytrid parasite-diatom host interactions from saline, brackish, and marine environments (Table 1).

Following Kohlmeyer and Kohlmeyer (1979) a lull occurred (Wei et al., 2010), and marine zoosporic fungi did not receive much work until 2009, when Hanic et al. documented a chytrid and oomycete infecting *Pseudo-nitzschia pungens* (Grunow ex Cleve) G.R.Hasle from Prince Edward Island in Canada; however, the chytrid was only seen once, and not identified to species. Other work, following diatoms, or other marine algae, did occur in the time window, but was not necessarily related to parasitism. In 2010, Wei et al. published “Oomycetes and fungi: important parasites on marine fungi,” which, while focused primarily on Oomycetes, did report 6 parasitic kingdom fungi, 5 of which were chytrids; however, none of these parasitized diatoms.

In 2011, Gleason et al. released “Zoosporic true fungi in marine ecosystems: a review,” which identified only 3 species of zoosporic true fungi that had been examined in any detail: *Rhizophydium littoreum* Amon, *Thalassochytrium gracilariopsis* Nyvall, Pedersen et Longcore and *Chytridium polysiphoniae* Cohn. Gleason et al. (2011) did identify more species that may fit into this category, but they did not make that distinction at that time; similarly, those “others” had no molecular data, and extremely limited morphological, habitat, host, and distribution data. Overall the authors remarked on the need for further study in this area. In 2014, Carney and Lane re-emphasized the lack of knowledge surrounding marine fungi, and framed it through the biofuel lens, as most diatoms, and algae in general, used to produce fuel are marine. When taking into account the known freshwater parasites, any extrapolation to marine life presents a serious economic risk for these systems and raises concerns regarding their viability.

Since 2016 a number of articles regarding marine systems and fungi have been released. Cleary et al. (2016), while focused on protists, and not fungi or diatoms, revealed that parasitic organisms could be much more prominent in marine environments than previously known. Gutiérrez et al. (2016) was the first report of diatoms parasitized by fungi in a productive coastal upwelling system. The diatoms, *Skeletonema* Greville*, Thalassiosira* Cleve and *Chaetoceros* Ehrenberg were found with chytrid sporangia attached. No chytrid species are identified, likely as little work has been done to characterize marine Chytridiomycota, but the authors do report that the sporangia appear host specific, and to coincide in density with the diatoms. Additionally, no comments on epidemics are made, but the authors do indicate that this means fungi likely play a part in the organic carbon cycle, a role for which diatoms are featured heavily (Smetacek, 1999; Smetacek, 2018).

Building on Gutiérrez et al. (2016), Garvetto et al. (2019) identifies a novel chytrid from the Rhizophydiales that parasitizes the host diatom *Skeletonema* sp. Garvetto et al. (2019) do not formally describe this chytrid, but they do provide comments on its morphology, and a molecular identification and phylogenetic placement. This is the most detailed account of a singular chytrid that infects a diatom from a marine habitat. Garvetto et al. (2019) also used the Ocean Sampling Day metabarcoding data set to determine a global distribution for this novel species, and found it was present on both sides of the Atlantic, and identified reads from closely related taxa present across the European coast. They end their article by pointing out the lack of information surrounding parasitism of diatoms in marine settings, especially in comparison to predation, which is currently understood to drive population dynamics. Kilias et al. (2020) also used the Ocean Sampling Day data to evaluate the role of diatoms as hosts for chytrids.

In the arctic, Hassett & Gradinger (2016) reported that chytrids have an unexpectedly large and undescribed biodiversity, and that they dominate the fungal population there. They also discuss changing light regimes as sea ice thins, and suggest chytrids could greatly alter phytoplankton populations in the future due to these changes. Kilias et al. (2020), examining co-occurrence of diatoms and chytrids, reported positive correlations with pennate diatoms, though these communities had relatively small populations, so the results are tenuous. The centric diatom *Thalassiosira* was also more dominant in these environs, indicating some level of host specificity occurring towards pennates. Kilias et al. (2020) hypothesized that these co-occurrences were being driven by melting ice water, and the corresponding biotic and abiotic changes, namely reduced salinity, so the parasitized species may not be “marine” in the sense of Kohlmeyer & Kohlmeyer (1979). Similar to Hassett & Gradinger (2016), Kilias et al. (2020) discuss the effects thinning sea ice may have on chytrid-diatom interactions, as it has been shown to potentially increase infection rates due to light stress in the host. In all, they posit that continued melting conditions may lead to increased parasitism, and that this is an area needing wider study.

Scholz et al. (2017b) reports some of the only experimental results exploring chytrid parasites infecting marine diatoms. Specifically, they examined the impact temperature, salinity, photon fluence rates, and differing photoperiods had on the infection susceptibility of diatom hosts from the genera *Navicula*, *Nitzschia* Hassal, *Rhizosolenia* Brightwell and *Chaetoceros.* Each host showed varying responses to the manipulated factors in regards to susceptibility of infection. An increase in proline, the indicator used to determine biological stress, was correlated with increased infection, and so was increased host densities.

The use of molecular methods to study chytrids, diatoms, and their interactions in marine environments, as well as freshwater, is still being developed, with some recent studies making inroads in this field (Käse et al., 2021; Reñé et al., 2022). Metabarcoding has been among the more tested methods, with examination of Long Term Ecological Research successfully revealing known host–parasite interactions (Käse et al., 2021), and revealing the extent to which these organisms comprise marine communities (Reñé et al., 2022). However, identification of new chytrid diatom interactions using these methods is not possible at this time (Käse et al., 2021; Reñé et al., 2022).Indeed, chytrids are still reported as extremely difficult to identify from marine settings (Käse et al., 2021), and they are still understudied as a whole (Reñé et al., 2022). In addition, traditional metabarcoding methods are biased against so-called early diverging fungi (Reynolds et al., 2022), so their abundance in these environments is likely underestimated in studies using the most commonly used fungal marker, i.e., ITS, and the standard primers.

Additionally, monitoring reports investigating the impacts of pathogens, often chytrids, on marine diatoms have been published in the past 10 years (Scholz, 2015; Scholz et al., 2016b), while some proposals outline future research directions (Rasconi et al., 2022). These studies identify difficulties in tying host diatoms to chytrid parasites at lower levels of taxonomy. They also emphasize the risks a lack of knowledge in this area present to future human needs. Specifically, it is intended that future monitoring projects will explore and identify gaps in the zoosporic parasite database. As biofuels are increasingly employed to meet human energy needs, closing these gaps will likely be critical for identifying, preventing, and mitigating infections in commercial systems; the knowledge gained from these programs will also likely be beneficial for assessing natural systems. Similarly, broad studies indicating chytrids are major pathogens in the ocean exist in the literature (Comeau et al., 2016; Jephcott et al., 2016), but these are only introduced here as they are not specific to diatoms.

Scholz et al. (2016a) provides an excellent summary of zoosporic parasites (in general) in marine settings, and indicates that future study in this field is a “black box” just waiting to be opened. Additionally, Cleary and Durbin (2016), Christaki et al. (2017), Garvetto et al. (2018), and Buaya et al. (2021a,b) examine oomycete parasites of diatoms in marine environments, or current approaches to studying parasites in marine settings.

## Conclusion

Until this review a comprehensive list of all documented diatom-chytrid interactions did not exist. This resource will hopefully serve as a reference point for future researchers studying diatom-parasite interactions, the mycoloop, and global planktonic communities. We also document the great need for future work examining both a greater breadth of taxonomic diversity of parasites and hosts and a greater depth of experiments probing these intricate and often highly specialized interactions.

## Author contributions

AD: Writing – original draft, Writing – review & editing, Conceptualization, Data curation. CQ: Supervision, Writing – review & editing, Conceptualization.
